# Suppressing non-radiative decay of photochromic organic molecular systems in the strong coupling regime†

**DOI:** 10.1039/d2cp00774f

**Published:** 2022-07-14

**Authors:** Rafael C. Couto, Markus Kowalewski

**Affiliations:** Department of Physics, Stockholm University, Albanova University Center SE-106 91 Stockholm Sweden rafael.carvalho@fysik.su.se markus.kowalewski@fysik.su.se

## Abstract

The lifetimes of electronic excited states have a strong influence on the efficiency of organic solar cells. However, in some molecular systems a given excited state lifetime is reduced due to the non-radiative decay through conical intersections. Several strategies may be used to suppress this decay channel. The use of the strong light-matter coupling provided in optical nano-cavities is the focus of this paper. Here, we consider the *meso*–*tert*-butyl-4,4-difluoro-4-bora-3*a*,4*a*-diaza-*s*-indacene molecule (*meso*–*tert*-butyl-BODIPY) as a showcase of how strong and ultrastrong coupling might help in the development of organic solar cells. The *meso*–*tert*-butyl-BODIPY is known for its low fluorescence yield caused by the non-radiative decay through a conical intersection. However, we show here that, by considering this system within a cavity, the strong coupling can lead to significant changes in the multidimensional landscape of the potential energy surfaces of *meso*–*tert*-butyl-BODIPY, suppressing almost completely the decay of the excited state wave packet back to the ground state. By means of multi configuration electronic structure calculations and nuclear wave packet dynamics, the coupling with the cavity is analyzed in-depth to provide further insight of the interaction. By fine-tuning the cavity field strength and resonance frequency, we show that one can change the nuclear dynamics in the excited state, and control the non-radiative decay. This may lead to a faster and more efficient population transfer or the suppression of it.

## Abstract 2

Strong and ultrastrong coupling *via* nanostructures can be considered a tool for optimizing photochromic organic molecular systems for solar cell applications.

## Graphical abstract

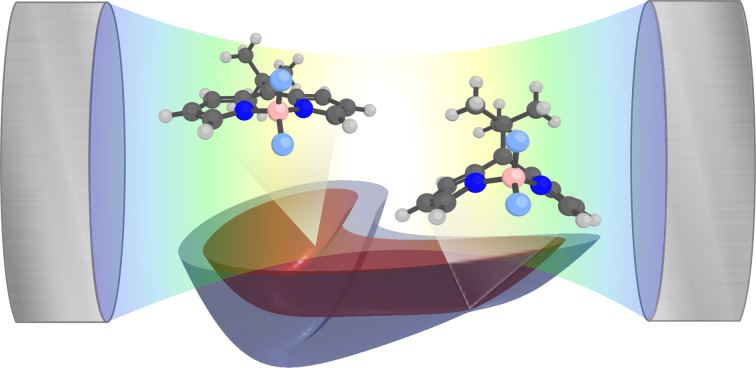

## 1 Introduction

The understanding of how a molecule in an electronic excited state behaves is one of the key components of the production of high efficient light harvesting photovoltaic cells. This can be related to spectral shape, range of absorption/emission, and the excited state lifetime, to name a few.^1,2^ A long-lived excited state in a photon acceptor molecule leads to a more efficient charge transfer for the energy conversion in solar cells. However, in some systems an ultrafast non-radiative decay from the electronically excited state, caused by the presence of a conical intersection (CoIn), is observed.^3^ There are several approaches to quench the non-radiative decay due to molecular dynamics in the excited state, which includes chemical substitutions, the use of high density or highly interactive solvents, to cite a few.^4–6^ Nonetheless, in the past years, it has been found that a potential energy surface's (PES) landscape might change significantly when a molecule is placed in an optical nano-cavity and strongly coupled to the quantized light field.^7–14^ This interaction creates polaritonic states, which are a combination of the PES of the bare molecule and the cavity field. By tuning the cavity properties, one can drastically change the wave packet evolution in such polaritonic PES.

**Fig. 1:**
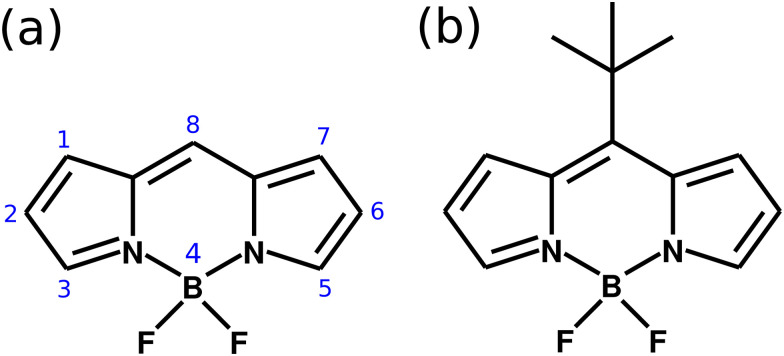
(a) Molecular structure of the BODIPY core, with the numbering showing the different substitution sites. (b) Structure of the *meso*–*tert*-butyl-BODIPY (MTBB).

**Fig. 2:**
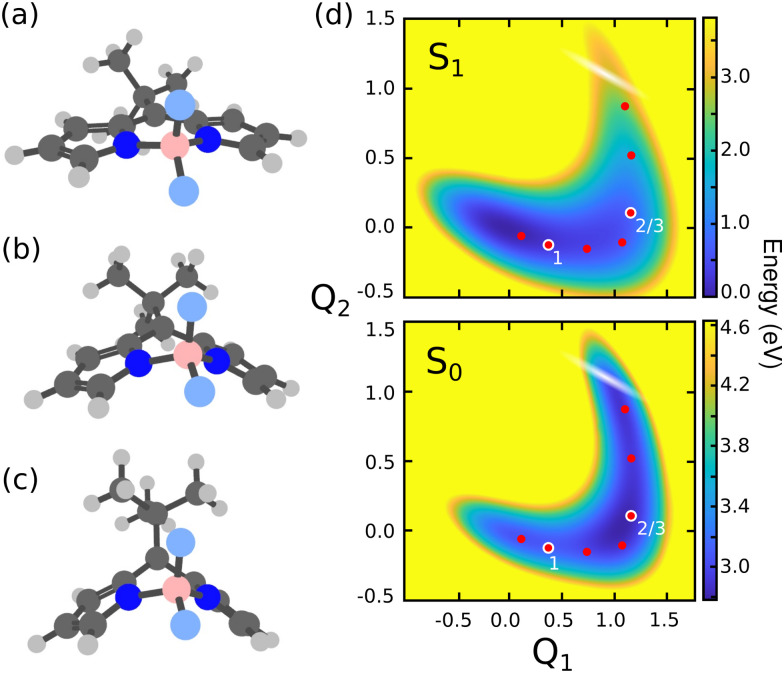
Molecular structures of the MTBB molecule at the (a) ground state equilibrium (S^eq^_0_), (b) S_1_ minimum (S^eq^_1_) and (c) conical intersection (CoIn). (d) Potential energy surfaces of along *Q*_1_ (S^eq^_0_ → S^eq^_1_) and *Q*_2_ (S^eq^_1_ → CoIn) coordinates for ground S_0_ and excited S_1_ states. The white shaded region represents the position of the CoIn seam and the dots show the position of the cavity frequencies chosen for the quantum dynamics simulations, while the numbering indicates the three cases referred in the discussion.

In this paper, we demonstrate theoretically how one can suppress the non-radiative decay of the wave packet in an electronic excited state when a molecule is coupled strongly to a cavity, by means of multi configuration electronic structure calculations and quantum nuclear wave packet simulations. The showcase system chosen is a derivative of the 4,4-difluoro-4-bora-3*a*,4*a*-diaza-*s*-indacene (also referred as BODIPY). This molecular system has being widely studied due to its optical properties with applications in organic solar cells.^15–20^ As presented in Fig. 1a, the core BODIPY structure has different substitution sites, covering various options to create derivatives with widely different properties. One very important aspect of BODIPY is the presence of a conical intersection in the HOMO–LUMO excited state (π → π* transition) which leads to a non-radiative decay of the excited state wave packet back to the ground electronic state. The essence of the excited state nuclear dynamics involves the bending of the central structure and the raise of the group at the position 8. In the case of the core BODIPY structure (Fig. 1a), there is a significant barrier between the Franck–Condon (FC) region and CoIn (around 2.9 eV), which makes the non-radiative decay irrelevant and the fluorescence yield of this system in around 0.99.^21^ However, different substitution groups may change drastically the fluorescence yield of the system. An example of this is the *meso*–*tert*-butyl-BODIPY (MTBB), depicted in Fig. 2b, which fluorescence yield is reported to be 0.04, due to the strong nuclear dynamics in the S_1_ electronic excited state.^6,22,23^

The low fluorescence yield of the MTBB molecule is the main motivation of the study presented here, with our goal being to direct the nuclear dynamics in the excited state in order to suppress the non-radiative decay of the wave packet back to the electronic ground state, creating a long-lived electronic excited state. To do so, this system is studied under the influence of strong light-matter coupling in a nano-cavity. We demonstrate that the cavity strong and ultrastrong coupling leads to a significant change in the PES landscape where the wave packet is trapped in the excited state due to the Rabi splitting between the polaritonic states, and the non-radiative decay is almost fully suppressed. There have been several experimental studies related to BODIPY in cavities, involving hybridization between the excitonic transitions,^24^ radiative pumping of polaritons,^25^ polariton condensation,^26^ polariton Lasing,^27^ anti-Stokes fluorescence^28^ and general optical properties.^29^ The main focus of this paper is on the suppression of the non-radiative decay of the wave packet in the excited electronic state.

## 2 Theory and model

### 2.1 MTBB structure and reaction coordinates

There are several possible derivatives of the BODIPY molecule, considering different substitution sites (Fig. 1a) present in the core structure. As the main goal of this paper is to understand the effects of a cavity in the excited state dynamics of BODIPY, a structure which presents a very low fluorescence yield, the MTBB (Fig. 1b and 2), was chosen. This molecule has been the subject of theoretical and experimental studies^6,22,23^ due to its characteristic low fluorescence yield related to strong non-adiabatic dynamics in the electronic excited state, leading to the non-radiative decay to the ground state through conical intersections.

The MTBB molecule possesses 99 nuclear degrees of freedom. To be able to treat this system with a full quantum wave packet description, a careful selection of the degrees of freedom has to be made. Through geometry optimization and gradient analysis of the ground and excited state, three critical structures were identified, which are presented in Fig. 2. The first one is the ground state equilibrium geometry (S^eq^_0_), which shows a relatively planar core structure, with the *tert*-butyl group rotated (Fig. 2a). The second structure (Fig. 2b) is the S_1_ excited state minimum (S^eq^_1_), characterized by a bent structure and the *tert*-butyl group rotated and symmetric to the molecule. The third important structure was identified at the point of the CoIn (Fig. 2c), displaying a more pronounced bent structure and the *tert*-butyl group with a ∼120° angle relative to the core of the molecule. Experimental and theoretical evidence^6,22,23^ shows that the low fluorescence yield is mainly caused by the non-radiative decay through the CoIn. Thus, the most probable reaction pathway of MTBB in the excited state involves the three molecular structures in Fig. 2a–c. Following this idea, the multidimensional MTBB system can be reduced to a 2D problem, with the reaction coordinates *Q*_1_ and *Q*_2_ defined as the difference between the Cartesian coordinates *x* of these molecular structures:1
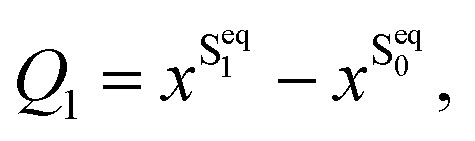
2
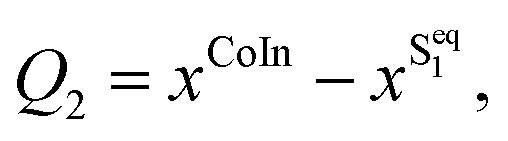
A similar approach has been successfully employed before.^30^ The PES represented by the reaction coordinates *Q*_1_ and *Q*_2_ are shown in Fig. 2(d). As can be seen, the PESs shows a “L” shape, going from the S^eq^_0_ to the CoIn position. The details of the PES will be discussed in the Results section.

### 2.2 Hamiltonian

A full quantum wave packet dynamics approach is used to understand the behavior of MTBB in the strong coupling regime. The Hamiltonian describing the light-matter interaction is defined as

3 *Ĥ*_MC_ = *Ĥ*_M_ + *Ĥ*_C_ + *Ĥ*_I_,

and is the sum of the Hamiltonian for the molecule (*Ĥ*_M_), cavity (*Ĥ*_C_) and the interaction between cavity and molecule (*Ĥ*_I_). A kinetic energy operator in *G*-matrix representation^31–33^ is used to take into account the non-orthogonal reaction coordinates *Q*_1_ and *Q*_2_. The molecular Hamiltonian reads4
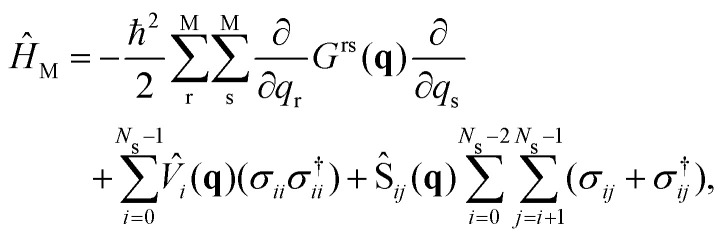
where the first term represents the kinetic energy operator in the *G*-matrix form, *V̂*_*i*_ the *i*th PES of the electronic states considered in the dynamics *N*_s_, Ŝ_*ij*_ the non-adiabatic coupling between electronic states S_*i*_ and S_*j*_ (here we consider S_0_ and S_1_ electronic states only) and *σ*_*ij*_ = |*i*〉〈*j*| and *σ*^†^_*ij*_ = |*j*〉〈*i*| are the electronic excitation creation and annihilation operators, respectively. The reaction coordinates are represented by **q** = (*Q*_1_, *Q*_2_)^*T*^. The *G*-matrix elements *G*^rs^ in eqn (4) take the form5
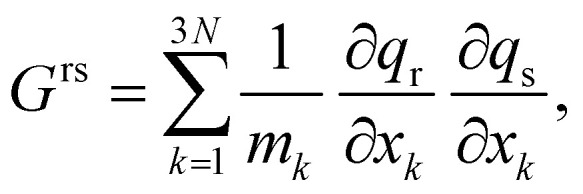
and act as generalized reduced mass, connecting the reaction coordinates **q** and atomic Cartesian coordinates *x*. Here, *N* is the number of atoms and *m*_*k*_ the mass of atom *k*. The inverse of *G* was computed for practical reasons^31–33^ and a root-mean-square deviation procedure using the Kabsch algorithm^34,35^ was employed to assure the Eckart conditions were fulfilled.^32,36^

The cavity Hamiltonian (eqn (3)) is defined as6
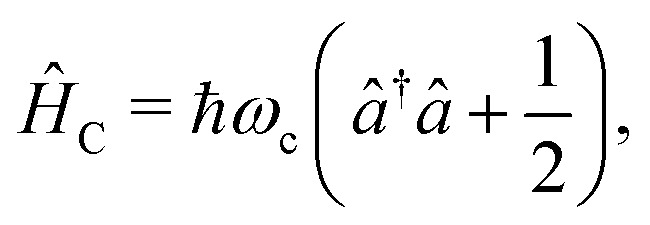
where *ω*_c_ is the cavity resonance frequency and *â* and *â*^†^ the photon mode creation and annihilation operators, respectively. Lastly, the description of the cavity-molecule interaction is given by^37^7
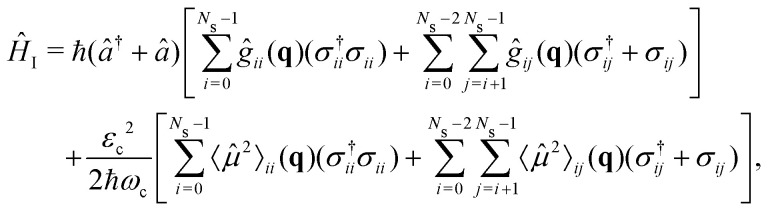
where *g*_*ij*_ are the cavity couplings between the electronic states *i* and *j*, given by the vacuum Rabi frequency8
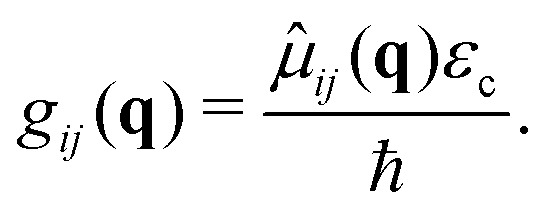
Here, *μ̂*_*ij*_ are the permanent (*i* = *j*) and transition (*i* ≠ *j*) dipole moments.〈*μ̂*^2^〉_*ij*_ = 〈*i*|*μ̂*^2^|*j*〉 is the squared dipole operator, describing the influence of the dipole self-energy interaction.^38^
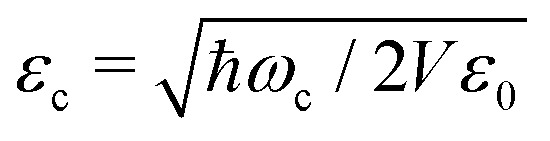
 is the cavity vacuum electric field strength with cavity mode volume *V*. Note that in eqn (7), the first term represents the electronic *g*_*ij*_ and vibrational *g*_*ii*_ cavity couplings, while the second term (dependent on 〈*μ̂*^2^〉_*ij*_) relates to the dipole self-energy interaction. The latter has shown to be important in the description of the light-matter interaction in complex systems.^37,39–41^

In order to treat the studied system in a numerical efficient way, we use photon displacement coordinates to describe the cavity mode (6) and (7).^9,37,42^ This is done by expressing the cavity mode's ladder operator as9
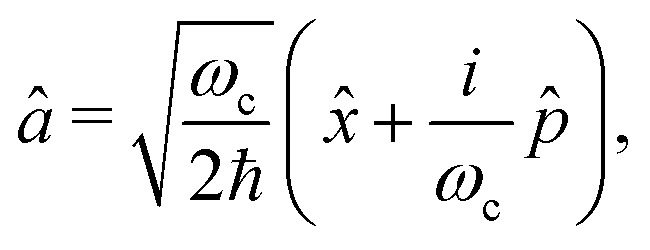
in terms of the photon displacement coordinate *x̂* and the conjugate momentum 
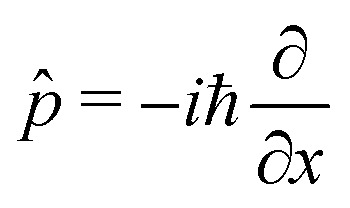
. Therefore, the cavity Hamiltonian (6) reads10
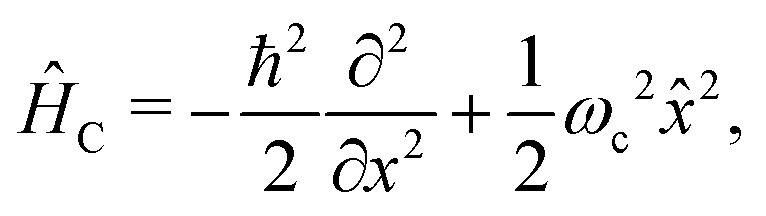
and the light matter interaction (eqn (7)) term takes the form11
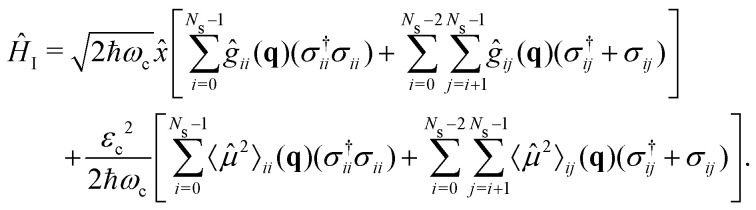
Note that this transformation preserves the counter-rotating terms *â*^†^*σ*^†^ and *âσ* in eqn (7).

## 3 Computational details

### 3.1 Electronic structure

The S_1_/S_0_ CoIn is responsible for the non-radiative decay back to the ground state. Thus, one could consider only the first two states in the quantum dynamics simulations. However, under the influence of ultrastrong coupling, higher lying electronic states may start to mix with the S_0_ and S_1_ and influence in the change in the PES landscape. Due to this, we considered in our simulations three upper electronic excited states, S_2_, S_3_ and S_4_ (see Fig. S2–S4 of ESI†). Higher lying states were neglected due to small transition dipole moment and large excitaton energies.

The PESs presented in Fig. 2d, and higher electronic states (see Fig. S2–S4 of ESI†), were computed with the state-average complete-active-space self-consistent-field (SA-CASSCF) method,^43–45^ with the cc-pVDZ basis set.^46^ The active space chosen consists of 12 electrons distributed over 11 orbitals, CAS(12,11), corresponding to the full set of the ππ* molecular orbitals (see Fig. S1 of ESI†). The molecular structures of the S_0_ and S_1_ minima (Fig. 2a and b) were obtained through geometry optimization with the OpenMolcas software,^47^ using the active space and basis set described above. The molecular structure at the CoIn (Fig. 2c) was obtained by the conical intersection optimization procedure implemented in the MOLPRO-2019 program package,^48,49^ with the same active space but the 6-311G* basis set.^50^ The transition dipole moments between all involved electronic states were computed in the velocity gauge with the restricted-active-space state-interaction approach^51,52^ and the non-adiabatic couplings between S_0_ and S_1_ were computed analytically,^53^ both implemented in the OpenMolcas software.

The analytical non-adiabatic couplings computed with OpenMolcas^47,53^ are represented by a 3*N* matrix (*N* is the number of atoms), related to the Cartesian coordinates of the atoms. It takes the form 〈*ψ*_*i*_|∂/∂*x*_*k*_*ψ*_*j*_〉, where *ψ*_*n*_ represents the wave function of the corresponding electronic state and *x*_*k*_ the Cartesian coordinate (*x*, *y*, *z*) for atom *k*. To convert the non-adiabatic coupling matrix from Cartesian coordinates to the reaction coordinates *Q*_1_ and *Q*_2_, the following transformation is used:^54^12
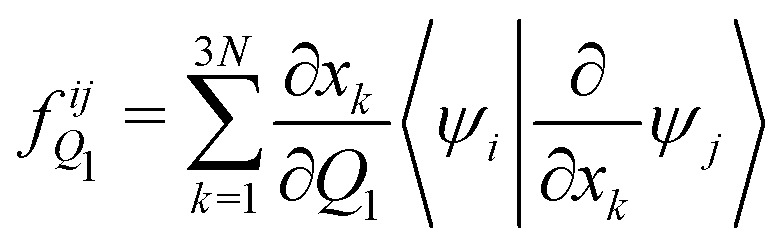
13
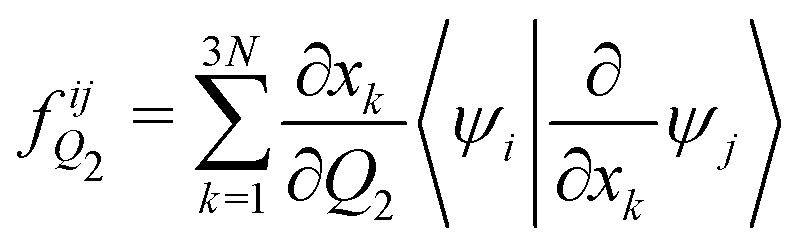
The last terms of eqn (12) and (13), 〈*ψ*_*i*_|∂/∂*x*_*k*_*ψ*_*j*_〉 are the non-adiabatic coupling terms in Cartesian coordinates and are taken directly from the OpenMolcas calculation. Note that the derivative ∂*x*_*k*_/∂*Q*_1/2_ is the same one used to obtain the inverse of the *G*-matrix (5). The calculated non-adiabatic coupling matrix elements show an arbitrary sign change, as the phase of the electronic states is also arbitrary.^53^ To correct this sign, a numeral continuity procedure was employed over the 2D surface, through both *Q*_1_ and *Q*_2_ coordinates.

The PES of all electronic states (Fig. 2d and Fig. S2–S4, ESI†), as well as all other properties (permanent and transition dipole moments, non-adiabatic coupling and *G*-matrix elements) were computed on a 43 × 33 (*Q*_1_, *Q*_2_) grid, which later were interpolated with a polyharmonic spline procedure^55^ to a 256 × 256 grid, with *Q*_1_ ranging from −1.0 to 1.75 and *Q*_2_ from −0.5 to 1.5. The permanent and transition dipole moments, *G*-matrix elements and non-adiabatic couplings used in the wave packet propagation are presented in Fig. S5–S22 of the ESI.†

### 3.2 Quantum nuclear wave packet dynamics

The excited state dynamics of MTBB was simulated with the quantum nuclear wave packet propagation method, where the time-dependent Schrödinger equation is solved numerically, considering the total Hamiltonian (3), which includes eqn (4), (10) and (11). The wave packet propagation is done by using the Chebychev method,^56^ implemented in our in-house code (QDng), with a maximum propagation time of 450 fs and a time step of 0.3 fs. Due to the use of photon displacement coordinate *x̂* (see eqn (10) and (11)), the dynamics were run on a 3D grid (256 × 256 × 32). Here, the last coordinate corresponds to the photon displacement coordinate *x̂*. The excited state dynamics is initiated by placing the ground state *ν*_0_ vibrational wave function on the S_1_ PES at the Franck–Condon region (*Q*_1_ = *Q*_2_ = 0.0) The vibrational ground state has been obtained by the imaginary time propagation method,^57^ also implemented in QDng.

Note that in eqn (11), we considered the interaction between all states involved, *i.e.*, the transition dipole moments between all electronic states: *μ̂*_01_,*μ̂*_12_,*μ̂*_34_…, with a total of 9 terms. The expectation value of the squared dipole operator 〈*μ̂*^2^〉_*ij*_(**q**) is computed by using the resolution of identity^37,41^14
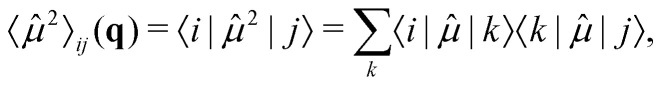
where *μ* is the dipole moment operator and *k* runs over all electronic states, *i.e.*, *k* = {0,1,2,3,4}.

The dynamics in presence of the cavity was simulated by considering two main external parameters, the cavity vacuum electric field strength *ε*_c_ and the cavity's resonance frequency *ω*_c_. To obtain an extended picture of the effect of the cavity coupling on the excited state dynamics, the chosen *ε*_c_ ranges from 0.005 to 2.06 GV m^−1^. The resonance frequency *ω*_c_ was set as the energy difference between the S_0_ and S_1_ PES at different points, following the excited state reaction coordinate which corresponds to the minimum energy pathway from the FC position to the CoIn in the S_1_ PES (see red dots in Fig. 2d). Therefore, *ω*_c_ ranges from 3.02 to 0.45 eV.

## 4 Results

### 4.1 Potential energy surfaces

The use of reactive coordinates requires a careful selection of the degrees of freedom considered in our simulations. Experimental evidence shows a fluorescence yield of around 0.04,^6^ meaning that almost all S_1_ population decays non-radiatively to the S_0_ PES. Our simulations for the bare molecule show a decay of ∼27% of the S_1_ wave packet through the CoIn. To understand this difference, a closer look into the wave packet propagation and the PES of MTBB (Fig. 2d) is required. In the excited state dynamics, the wave packet in the S_1_ PES would travel from the FC region (*Q*_1_ = *Q*_2_ = 0.0) along the *Q*_1_ coordinate until *Q*_1_ ∼ 1, which takes around 120 fs. Here, part of the wave packet returns to the FC region due to the presence of barrier at around *Q*_1_ = 0.5 (*Q*_2_ = 0.0), of about 0.025 eV. Even though this barrier is relatively small, the wave packet does not have enough momentum to fully overcome it and only part of it reaches *Q*_1_ ∼ 1. Furthermore, after ∼120 fs, the wave packet continues along the *Q*_2_ coordinate, reaching the CoIn region at *t* ∼ 200 fs. Here, the presence of another barrier (*Q*_1_ ∼ 1, *Q*_2_ ∼ 0.5), of about 0.13 eV, slows down the wave packet. The appearance of these two barriers and the fact that the wave packet propagation is limited to 500 fs are the main factors that lead to a ∼27% non-radiative decay.

The two small barriers seen in the S_1_ PES can be understood through the geometry optimization of the S^eq^_1_ and CoIn structures (Fig. 2b and c), which roughly shows the minimum energy pathway to get to these structures. Starting from the S^eq^_0_, it was observed that the MTBB molecule bends the core first and then rotates the *tert*-butyl group (Fig. 2b), which would lead to a barrier less potential. However, in our selected reaction coordinate *Q*_1_, the bending and rotation processes happen simultaneously, and a small barrier is observed. The same can be said about the CoIn structure (Fig. 2c), where our simulations show a sequential movement of the bending the core and raising the *tert*-butyl group, while the *Q*_2_ represents this motion concurrently.

We acknowledge the difference between the experimental results and our simulations in fluorescence yield and focus on the relative changes that occur in the presence of the cavity.

### 4.2 Dynamics in the strong coupling regime

**Fig. 3:**
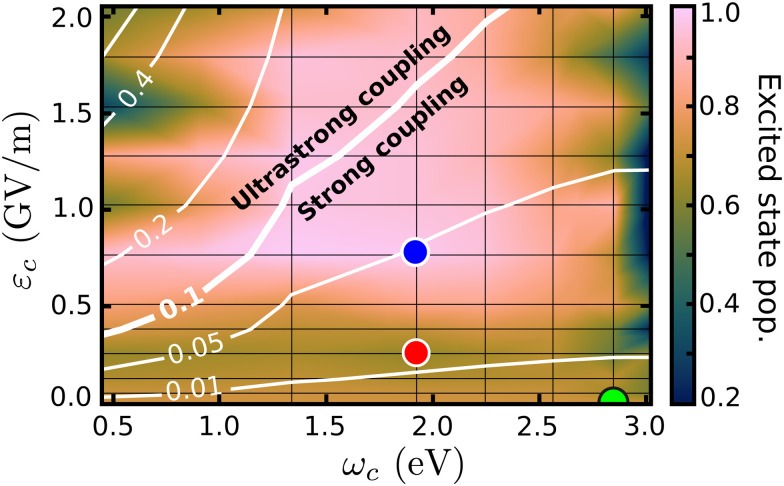
Population in the excited state, at 400 fs, as a function of the cavity resonance frequency *ω*_c_ and field strength *ε*_c_. The coupling strength (*g*_*ij*_/*ω*_c_) is shown by the contour lines. The colored dots mark the position of the cases considered in Fig. 4 and in the discussion.

The cavity quantum dynamics of the MTBB excited state was simulated and the field strength *ε*_c_ and the resonance frequency *ω*_c_ have been varied. The results are presented in the form of a 2D map in Fig. 3, which shows the total population in the electronic excited state at 400 fs. This propagation time was chosen as it represents the maximum population decay (within our simulations), whereas after this point the wave packet travels back and forth in the PES. Alongside the population, a contour map of the coupling strength (*g*_*ij*_/*ω*_c_) is shown. It is assumed that the ultrastrong coupling regime is when *g*_*ij*_/*ω*_c_ > 0.1^58–60^ is reached, as indicated in Fig. 3.

The wave packet propagates exclusively in the S_1_ and S_0_ PESs for cavity coupling strengths *g*_*ij*_/*w*_c_ < 0.1. However, in the ultrastrong coupling region, a small portion of the population (up to 10% in the region with *g*_*ij*_/*w*_c_ > 0.4) is projected to higher excited states (S_2_, S_3_ and S_4_). Due to this mixing, the time evolution of the populations presented in this paper is related to the total population in the excited states and includes S_1_ to S_4_. The 2D map shows significant changes in the final excited state populations when varying *ε*_c_ and *ω*_c_, from suppression to increase of the non-radiative decay. On the right-most side of Fig. 3, the resonance frequency is set to the FC region, with leads to strong Rabi oscillations (see Fig. S8 in the ESI†), where at 400 fs shows population around 0.2. When considering the extreme case, with the smallest *ω*_c_ = 0.45 eV and *ε*_c_ > 1.3, the excited state population is transferred almost completely to the electronic S_0_ ground state. Moreover, let us focus on the region within the extremes and analyze three cases that illustrate the major effects of the strong coupling in the excited state dynamics of MTBB.

Fig. 3 presents three main regions related to the influence of the strong coupling. The first region represents the weak cavity field interaction, close to *ε*_c_ = 0.0, where the excited state population remains at around 73%, as for the bare molecule. With the increase of the field strength (*ε*_c_ ∼ 0.25 GV m^−1^), a decrease of the final excited state population of up to 62% is observed. The third region relates to the suppression of the non-radiative decay, observed at approximately *ε*_c_ > 0.8 GV m^−1^. In all three regions, it is also observed that the cavity resonance frequency plays an important role in the excited state dynamics. The intricacies of these different effects will be discussed in the following.

**Fig. 4:**
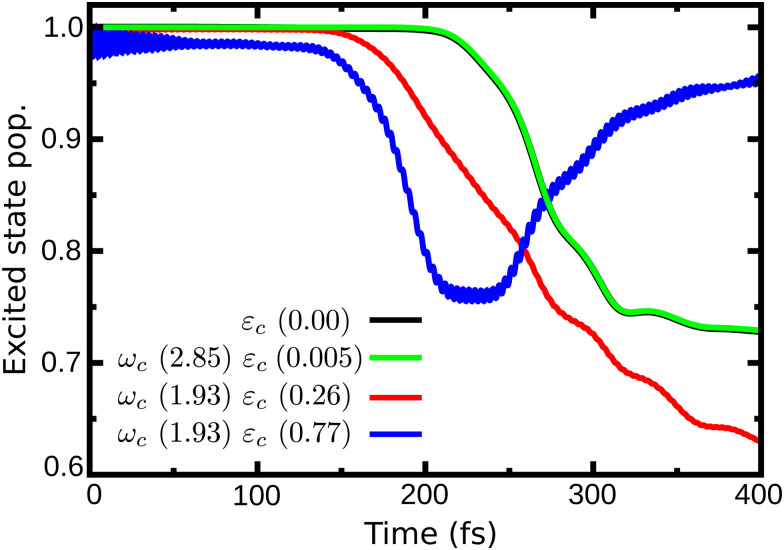
Time dependent population in the excited state for case (1) (*ω*_c_ = 2.85 eV, *ε*_c_ = 0.005 GV m^−1^), case (2) (*ω*_c_ = 1.93 eV, *ε*_c_ = 0.26 GV m^−1^) and case (3) (*ω*_c_ = 1.93 eV, *ε*_c_ = 0.77 GV m^−1^).

In Fig. 4 are presented three cases that represent the major cavity effects in the excited state nuclear dynamics. The time-dependent excited state population for case (1), *ω*_c_ = 2.85 eV, *ε*_c_ = 0.005 GV m^−1^ (representing the minimum cavity effect), case (2), *ω*_c_ = 1.93 eV, *ε*_c_ = 0.26 GV m^−1^ (representing the well in Fig. 3), and case (3) – *ω*_c_ = 1.93 eV, *ε*_c_ = 0.77 GV m^−1^ (illustrating the suppression of the non-radiative decay). All three cases are marked in Fig. 4 with color-coded circles. The results for the bare molecule (*ε*_c_ = 0.00) are shown for comparison and the positions of the resonance frequency in the PES are shown in Fig. 2b. In case (1), the cavity's resonance frequency is set close the FC region (*Q*_1_ ≈ −0.1 *Q*_2_ ≈ 0.4 – Fig. 2d), while the field strength is set to its lowest value considered here (*ε*_c_ = 0.005 GV m^−1^). This results in a minimum change of the population transfer to the ground state when compared to the bare molecule (green and black curves in Fig. 4, respectively). Case (2): by increasing the field strength and setting the resonance frequency at the barrier along *Q*_2_ of the S_1_ PES (*Q*_1_ ≈ 1.1, *Q*_2_ ≈ 0.1; Fig. 2d), an increase of the non-radiative decay can be seen (Fig. 4 red curve), with the excited state population reaching 0.62 at 400 fs. This constitutes a change of about 15% when compared to the bare molecule. In the third scenario, case (3), an interesting behavior is observed. As seen in the blue curve in Fig. 4, the beginning of the propagation is dominated by large Rabi oscillations, due to the strong coupled field strength considered (*ε*_c_ = 0.77 GV m^−1^), leading to a direct transfer of about 3% of excited state population to the ground state. After around 100 fs, a rapid decay to S_0_ is observed, with the excited state population reaching ∼0.75. However, at 200 fs, the population is transferred back to excited state, which at the end of the propagation (400 fs) is 0.95.

**Fig. 5:**
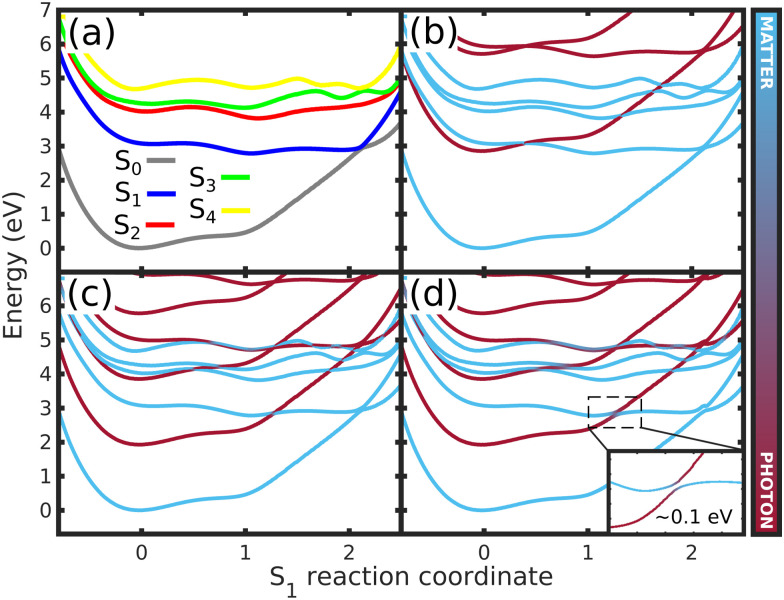
Potential energy curves along the S_1_ reaction coordinate (S_1,RC_) for (a) the bare molecule, (b) case (1) (*ω*_c_ = 2.85 eV, *ε*_c_ = 0.005 GV m^−1^), (c) case (2) (*ω*_c_ = 1.93 eV, *ε*_c_ = 0.26 GV m^−1^) and (d) case (3) (*ω*_c_ = 1.93 eV, *ε*_c_ = 0.77 GV m^−1^) – (Fig. 3 and 4). In (a), the curves represents the 1D cuts of the 2D PES (Fig. 2d and Fig. S2–S4, ESI†) of all electronic states. (b)–(d) shows the approximate polaritonic potential energy curves obtained from the diagonalization of the Hamiltonian (3) in the basis of the Fock states, considering |S_0_|0〉 to |S_4_|0〉 and up to three photons for S_0_ and S_1_ electronic states (|S_0_|1〉,|S_0_|2〉,|S_0_|3〉, …). The insert in (d) shows the Rabi splitting between upper and lower polaritons. The color bar represents the photon-matter interaction of the UP and LP states.

To shed light on how the cavity strong coupling changes the PESs landscape, we analyze the approximate 1D cuts of the polaritonics surfaces along the S_1_ reaction coordinate (Fig. 5a). The 1D reaction coordinate was chosen by considering the minimum energy pathway of the wave packet in the S_1_ PES, namely the S_1_ reaction coordinate (S_1,RC_). These 1D cuts show the main features of the S_1_ PES (Fig. 5): the FC region at position S_1,RC_ = 0.0, the barrier along the *Q*_1_ coordinate (S_1,RC_ ≈ 0.5), the S_1_ minimum (S_1,RC_ = 1.0), the barrier along *Q*_2_ (S_1,RC_ ≈ 1.5), and the CoIn (S_1,RC_ ≈ 2.0). The 1D polaritonic potential energy curves (PEC) were obtained by the diagonalization of the Hamiltonian in the basis of the Fock states (Fig. 5b–d), and considering |S_0_|0〉 to |S_4_|0〉, and S_0_ and S_1_ with up to three photon modes, *i.e.*, |S_0_|1〉, |S_0_|2〉, |S_0_|3〉…. Note that we have neglected the counter-rotating terms in the diagonalization, and the 1D polaritonic surfaces presented here are used for a qualitative understanding of the dynamics. All results presented in the paper were obtained from the full PES as described in Section 2.

**Fig. 6:**
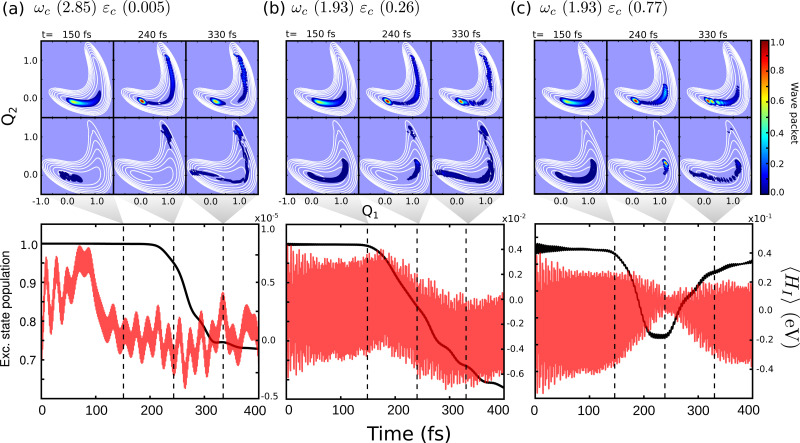
Time-dependent wave packet |*Ψ*(*Q*_1_,*Q*_2_)〉 in the S_0_ and S_1_ PES for the dynamics considering (a) case (1) – *ω*_c_ = 2.85 eV, *ε*_c_ = 0.005 GV m^−1^, (b) case (2) – *ω*_c_ = 1.93 eV, *ε*_c_ = 0.26 GV m^−1^ and (c) case (3) – *ω*_c_ = 1.93 eV, *ε*_c_ = 0.77 GV m^−1^. The bottom panels show the time-dependent excited state population and expectation value of the interaction Hamiltonian 〈*H*_I_〉 (eqn (7)). The dashed lines indicate correspondent time of the wave packet plots, 150, 240 and 330 fs.

Comparing the polaritonic 1D curves for case (1) (*ω*_c_ = 2.85 eV, *ε*_c_ = 0.005 GV m^−1^ – Fig. 5b) with the bare molecule (Fig. 5a), it becomes clear why the wave packet dynamics results are very similar. The strong coupling between the upper (UP) and lower (LP) polaritons can be seen close to the FC region, mainly related to |S_0_|1〉, as higher photon polaritons are above 6 eV. It leads to a free movement of the wave packet on the LP towards the CoIn through a merely altered PES, when compared to the S_1_ PEC of the bare molecule. For case (2), (Fig. 5c) the difference is more striking. Now, the crossing between UP and LP PECs appears at S_1,RC_ ≈ 1.3, which leads to a significant change in the dynamics. The excited state wave packet has to overcome two barriers (along *Q*_1_ and *Q*_2_) in order to reach the CoIn, slowing it down and even trapping it in certain regions of the PES. Moreover, by tuning the cavity resonance frequency in these regions and using moderate field strengths, the wave packet may overcome these barriers more easily, leading to an increase of the population transfer to the ground state PES. Case (2) (Fig. 5c), is an example that illustrates these phenomena, as the crossing between UP and LP appears in the region close to the barrier at S_1,RC_ ≈ 1.3. As in case (1), the potentials are mostly affected by |S_0_|1〉. A larger non-radiative decay is observed compared to the bare molecule and case (1). In Fig. 6 the time-dependent wave packet is shown in both S_0_ and S_1_ electronic states' PES along with the expectation value of the interaction Hamiltonian (eqn (7)) and the excited state population, for the three cases presented in Fig. 4 and 5. The wave packet |*Ψ*(*Q*_1_,*Q*_2_)〉 was obtained from the full 3D quantum dynamics, where the wave packet has been integrated over the *x̂* coordinate: 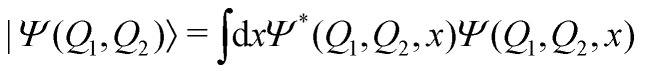. By comparing the panels (a) and (b) of Fig. 6, a similar wave packet propagation behavior can be observed: only part of the wave packet reaches the CoIn, while the majority is trapped close the FC region (*Q*_1_ ≈ 0.1/*Q*_2_ ≈ 0.0). However, case (2) (Fig. 6b) shows that, due to the strong cavity coupling close the barrier along *Q*_2_ of the S_1_ PES (*Q*_1_ ≈ 1.1/*Q*_2_ ≈ 0.1 or S_1,RC_ ≈ 1.3 in Fig. 5), the wave packet has a more direct pathway to reach the CoIn, leading to a bigger population transfer to S_0_ (increase of around 62%). Another striking feature is the significant increase in the cavity coupling strength (〈*H*_I_〉), of about three order of magnitude from case (1) (Fig. 5a) to case 2 (Fig. 5b). The case (2) (*ω*_c_ = 1.93 eV/*ε*_c_ = 0.26 GV m^−1^) represents the behavior throughout the lower population valley of Fig. 3, where in each case, the cavity's resonance frequency and field strength couples the regions of the PES related to the barrier along the *Q*_1_ coordinate (S_1,RC_ ≈ 0.5 of Fig. 5a), the S_1_ minimum (S_1,RC_ = 1.0), and the barrier along *Q*_2_ (S_1,RC_ ≈ 1.5), which facilitates the wave packet reaching to the CoIn region, leading to a bigger population transfer to S_0_.

Moving on to the last case of this analysis, with *ω*_c_ = 1.93 eV/*ε*_c_ = 0.77 GV m^−1^ (case 3), which represents the strong suppression of the non-radiative decay of MTBB excited state dynamics. The time-dependent excited state population (Fig. 4) shows an oscillatory behavior in the population between the excited states and S_0_. The polaritonic curves for this study case (Fig. 5d) shows a significant Rabi splitting (∼0.1 eV) between UP and LP PECs of low photon modes (S_0_|1〉), however, a population exchange still occurs between the two states. Let us analyze the time-dependent wave packet presented in Fig. 6c. First, the strong cavity coupling led to the most significant changes in the PES, as the wave packet does not reach the CoIn region, even though the same *ω*_c_ from case (2) is considered. At *t* = 150 fs, when the excited state population begins to decrease, the wave packet reaches the region where the UP and LP are close together. This also reduces the Rabi oscillation observed in the coupling strength 〈*H*_I_〉 (Fig. 6). However, due to the large Rabi splitting (≈0.1 eV) between the UP and LP PESs (Fig. 5d), the wave packet is never actually transferred to the S_0_ state. When the wave packet reaches this region and slows down (*t* = 240 fs), the large cavity coupling strength strongly couples the wave packet in both excited and ground electronic states. (Fig. 6c). Moreover, when the wave packet starts moving back on the PES (*t* = 330 fs), no significant population is actually transferred to the S_0_ electronic state and the wave packet is trapped in the excited state, suppressing the non-radiative decay. These phenomena can be seen throughout the region where the excited state population is greater than 0.80 (Fig. 3), with the main difference being the smaller coupling strength which leads to a less pronounced population change (see Fig. S8 in the ESI†).

The strong coupling with the cavity drastically changes the excited state nuclear dynamics, leading to the minimization of the non-radiative decay. The interaction Hamiltonian *H*_I_ (eqn (11)) is governed by the two terms that describe the electronic and vibrational cavity coupling, dependent on *g*_*ij*_, and the dipole self-energy, which depends on 〈*μ*^2^〉_*ij*_. In addition, each of these terms connects the transition and permanent dipole moments of the studied system. Thus, to have a better understanding of the importance of these terms, the cavity-coupled dynamics was simulated by using three modified versions of the Hamiltonian *Ĥ*_I_(11):15
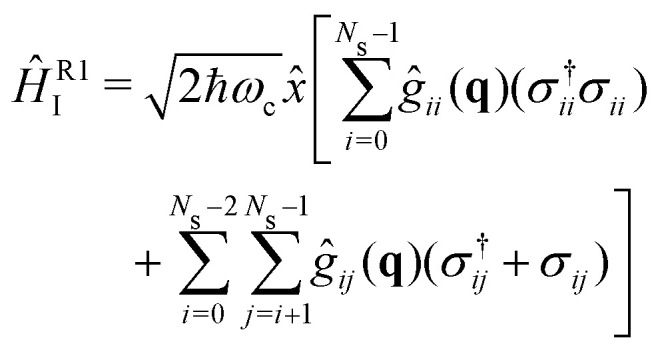
16
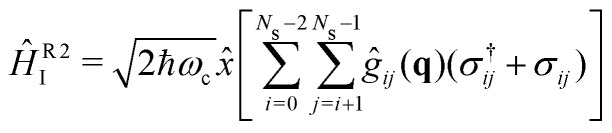
17
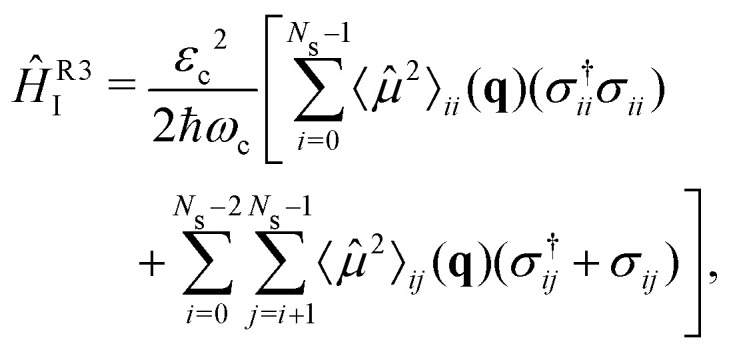


**Fig. 7:**
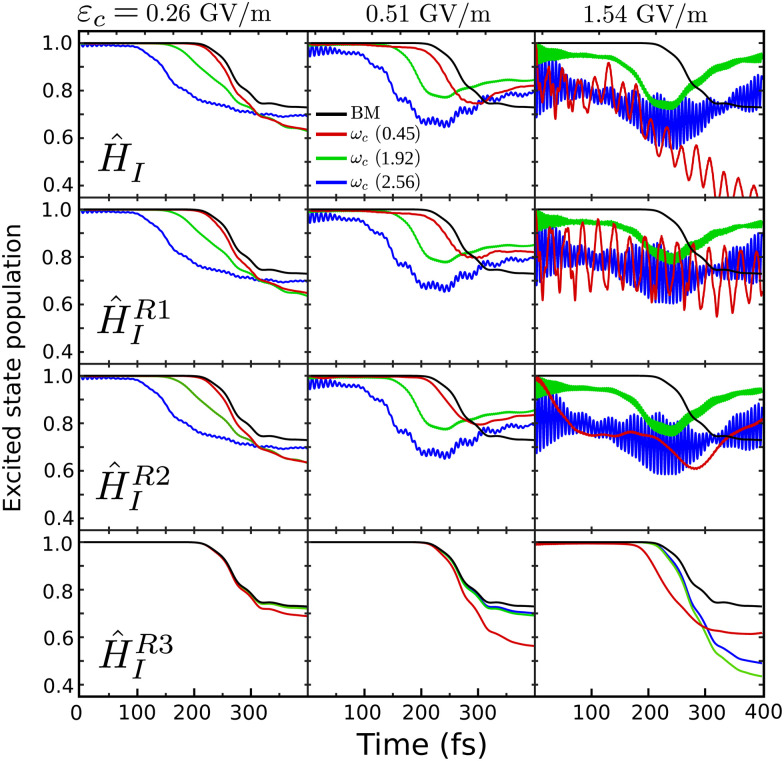
Influence of different terms of the interaction Hamiltonian *Ĥ*_I_ in the cavity coupled quantum dynamics. It is presented the results for *ε*_c_ = 0.26, 0.51 and 1.54 GV m^−1^, and *ω*_c_ = 0.45, 1.92 and 2.56 eV, as well as the bare molecule (BM). The time-dependent excited state population is shown for, from top to bottom, the full *Ĥ*_I_ Hamiltonian (11), the reduced *Ĥ*^R1^_I_(15), *Ĥ*^R2^_I_(16) and *Ĥ*^R3^_I_(17).

The first Hamiltonian (15) comprehends just the electronic *ĝ*_*i*_*j* and vibrational *ĝ*_*i*_*i* cavity coupling terms. In the second Hamiltonian (16), only the electronic states coupling *ĝ*_*i*_*j* is included. In the third Hamiltonian (17), the electronic and vibrational cavity coupling is neglected and only the dipole self-energy, dependent on 〈*μ*^2^〉_*ij*_, are considered. For this analysis, three values for the cavity field strength were chosen, *ε*_c_ = 0.26, 0.51 and 1.54 GV m^−1^, and three different resonant frequencies *ω*_c_, 2.56, 1.92 and 0.45 eV. In Fig. 7 is presented the time-dependent excited state population for the full interaction Hamiltonian (11) and the three reduced versions (eqn (15)–(17)), along with the bare molecule results for comparison. For the smaller field strength 0.26 and 0.51 GV m^−1^, it is observed that the main coupled dynamics behavior is related to the electronic states coupling term *ĝ*_*ij*_, as small changes in time-dependent population is seeing when comparing the results from *Ĥ*_I_, *Ĥ*^R1^_I_ and *Ĥ*^R2^_I_. It is also observed that self-dipole terms have small influence in the dynamics, becoming more significant for smaller resonance frequencies (*ω*_c_ = 0.45 eV). For the case with the strongest coupling (*ε*_c_ = 1.54 GV m^−1^), the effect of the different terms of *H*_*i*_ is quite evident, specially for *ω*_c_ = 0.45 eV. In this case, the self-dipole terms shown to be quite important, as pointed out by other authors.^37,39–41^

There are a few aspects that were not considered in the simulations of the excited state dynamics of MTBB in nano-cavities that should be addressed. The first one relates to the collective light-matter coupling, which has been shown to influence the excited state dynamics.^61–65^ The collective effects in molecular assembly have shown to have a significant effect on the photo dissociation mechanism, however, the size of the considered system can change drastically with this effect.^64^ On a molecular system, it was observed that the collective coupling effects are sensitive to the cavity resonance frequency when going from the FC to the CoIn,^65^ but also shown to be system dependent. Nevertheless, this effect might strongly influence the excited state dynamics of MTBB and to have a more complete description of the cavity effects in the non-radiative decay suppression, such terms would be needed to be taken into account.

The effects of a finite photon life on the dynamics have been neglected in this study. It should be noted that these effects can be quite significant for short-lived cavity modes and affect the dynamics in non-trivial ways.^30,60,66–69^ However, the size and type of the numerical Hamiltonian does not permit the use of Lindblad equation or the use of a simple non-Hermitian term. The results in this study thus need to be interpreted in the context of strong coupling with low photon loss rates. MTBB in lossy cavities will be the subject of future study.

## 5 Conclusions

The excited state nuclear dynamics of MTBB under the influence of strong and ultrastrong coupling has been simulated by means of multiconfigurational electronic structure calculation and quantum dynamical wave packet simulations. Through a careful selection of the reaction coordinates that represent the MTBB dynamics in the excited state, the multidimensional system was reduced to a 2D problem. This allowed for a full description of the light-matter interaction of MTBB in a strong coupling regime.

The 2D MTBB PES exhibits two barriers that act as a trap and/or slow the wave packet, making it less likely to reach the conical intersection. However, tuning of the cavity resonance frequency and field strength leads to significant changes in the potential energy landscape, effectively removing the barriers, and increasing the population transfer from the excited electronic state to the electronic ground state. This effect can be understood by analysis of the polaritonic potentials and the visualization of the time-dependent wave packets. This interesting feature might be used to identify transition states in multidimensional molecular systems, by monitoring the population change through the scan with different cavity parameters. When a faster decay is observed, it could indicate that the cavity is coupled to a region where a barrier is present, which can be directly related to a transition state structure.

We demonstrated that, by tuning the cavity parameters, population transfer between the electronic states can be increased or decreased. Considering the possibility of suppressing the non-radiative decay of the excited state, we have shown that the interplay between the cavity's resonance frequency and field strength is crucial. This can be done by carefully tuning both parameters, while reaching the ultrastrong coupling regime. In the cases where the non-radiative decay suppression is observed, the coupling is so strong that the wave packet is briefly projected onto the ground state PESs, even though the Rabi splitting between the upper and lower polaritons was ∼0.1 eV. With the focus in the suppression of the non-radiative decay, an optimal region is clearly observed, as the increase of the coupling strength led to a reduction of this effect, as well as tuning the cavity frequency to the FC region.

Other aspects of the strong coupled nuclear dynamics were analyzed, such as the influence of the different terms of the interaction Hamiltonian *Ĥ*_I_. It was shown that not only the electronic and vibrational coupling terms are important, but the dipole self-energy interaction terms are important for the suppression of the non-radiative decay.

Strong and ultrastrong coupling *via* nanostructures can be considered a tool for optimizing photochromic organic molecular systems for solar cell applications. By considering the showcased BODIPY structure, it was shown that ultrastrong coupling can drastically increase the fluorescence yield of such systems. This might open the field for the application of simpler and cheaper organic molecules in solar cell manufacturing, where the extension of the excited state lifetimes can be done using strongly coupled nano-cavities. This paper was focused on this one aspect, however, other important factors might be considered in the future, such as a shift in absorption and emission bands, efficiency of the charge transfer, as well as molecular design and optimization.

## Conflicts of interest

There are no conflicts to declare.

## Supplementary Material

CP-024-D2CP00774F-s001
