# Supplementary material for: Suppressing non-radiative decay of photochromic organic molecular systems in the strong coupling regime
Source: Phys Chem Chem Phys. 2022 Jul 14;24(32):19199–208. doi: 10.1039/d2cp00774f (PMC9382694; doi:10.1039/d2cp00774f)
Supplement: CP-024-D2CP00774F-s001 [file CP-024-D2CP00774F-s001.pdf]

Electronic Supplementary Material (ESI) for Physical Chemistry Chemical Physics.
This journal is © the Owner Societies 2022

Supplementary information: Suppressing non-radiative decay of photochromic organic molecular systems in the strong coupling regime

Rafael C. Couto* and Markus Kowalewski*

Department of Physics, Stockholm University, Albanova University Center, SE-106 91 Stockholm, Sweden

E-mail: rafael.carvalho@fysik.su.se; markus.kowalewski@fysik.su.se

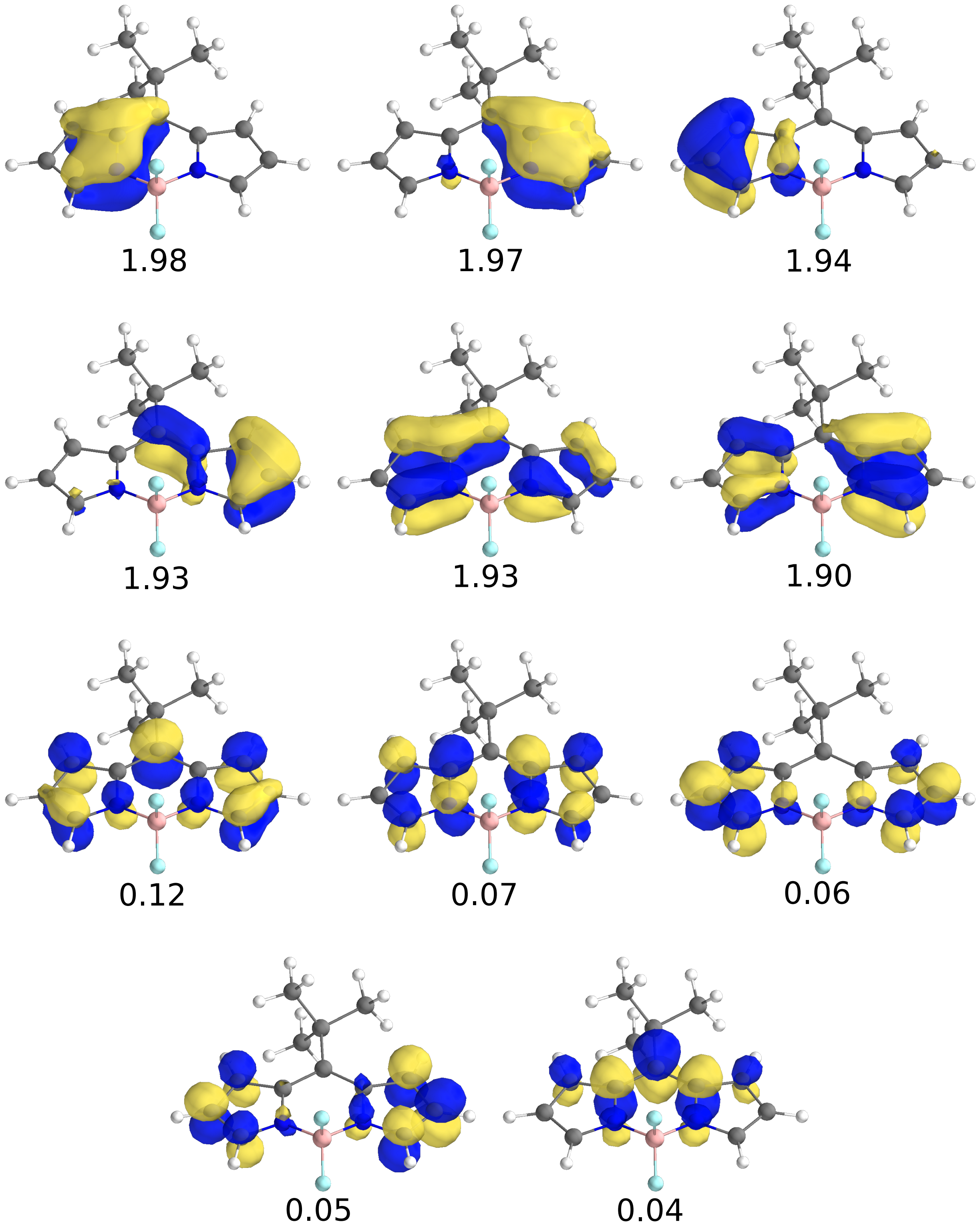

Figure S1: Molecular natural orbitals of meso-tert-butyl-BODIPY used in the CASSCF electronic structure calculations. Below the orbital plots is shown the orbital occupation in the ground electronic state.

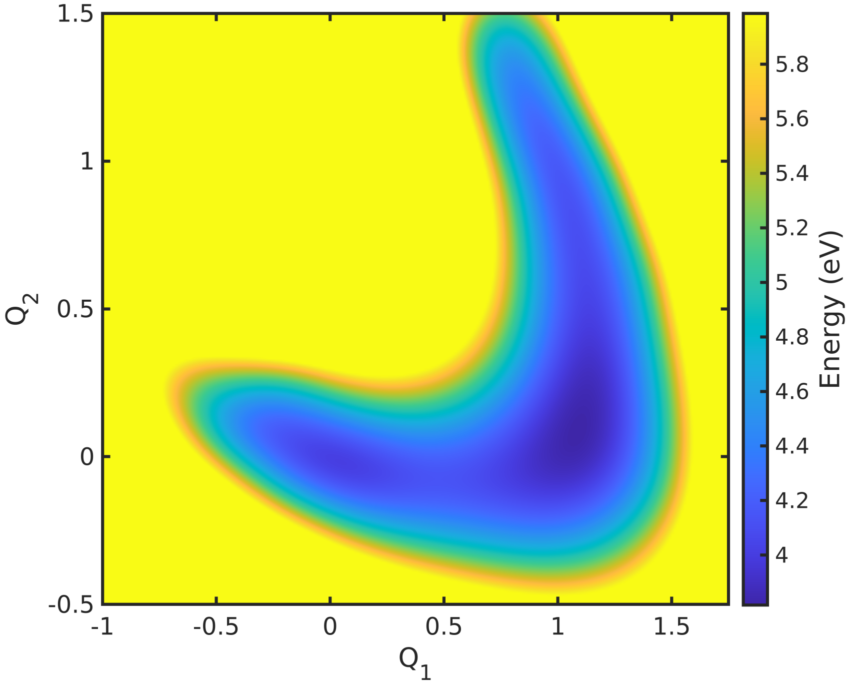

Figure S2: Potential energy surface of electronic state S_2.

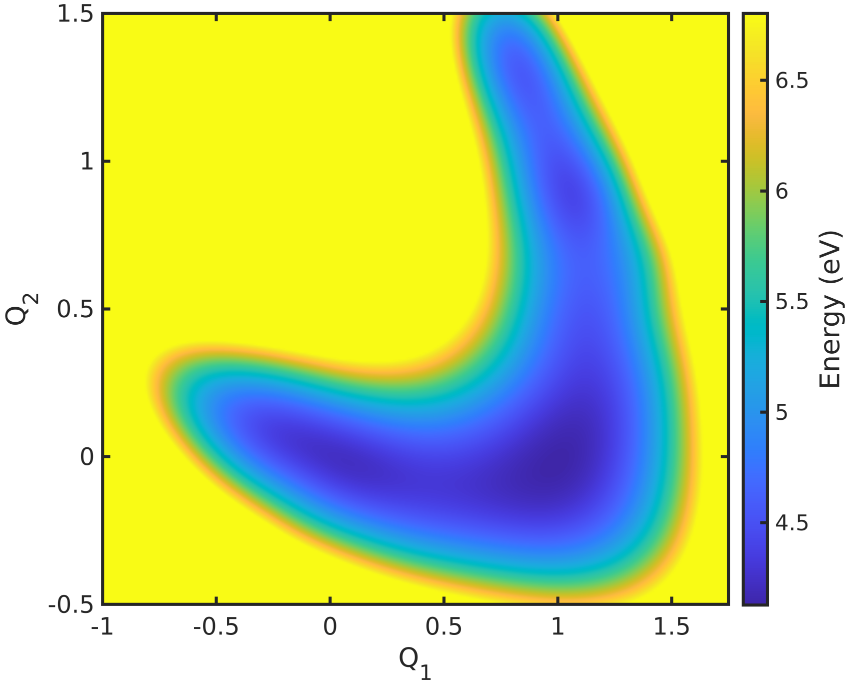

Figure S3: Potential energy surface of electronic state S_3.

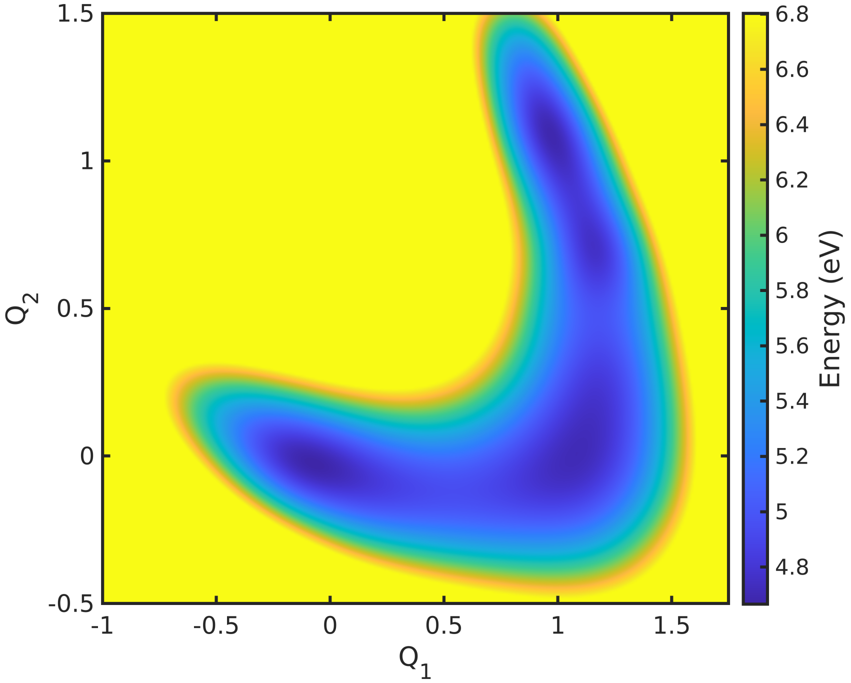

Figure S4: Potential energy surface of electronic state S_4.

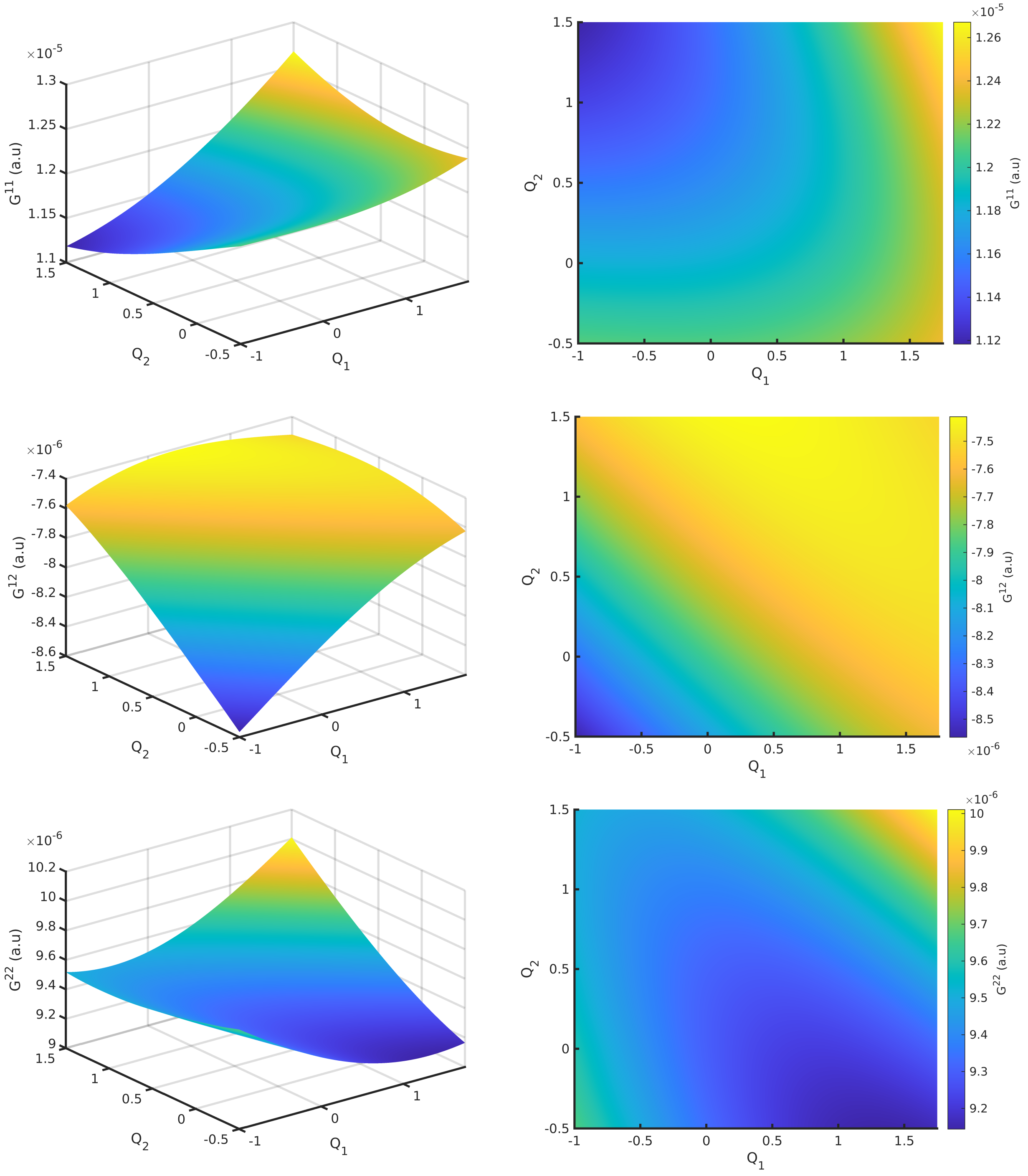

Figure S5: G-matrix elements.

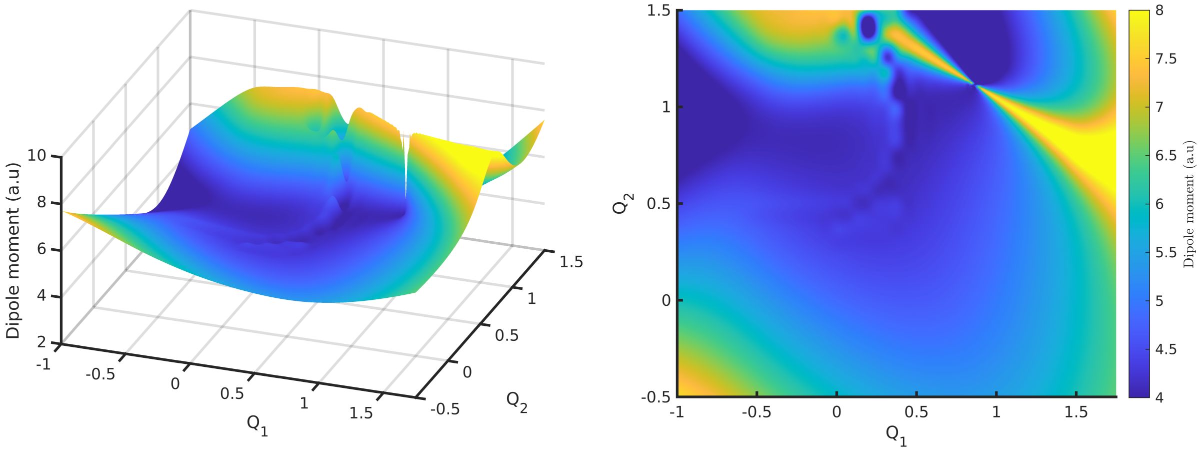

Figure S6: Permanent dipole moment of ground electronic state S_0 .

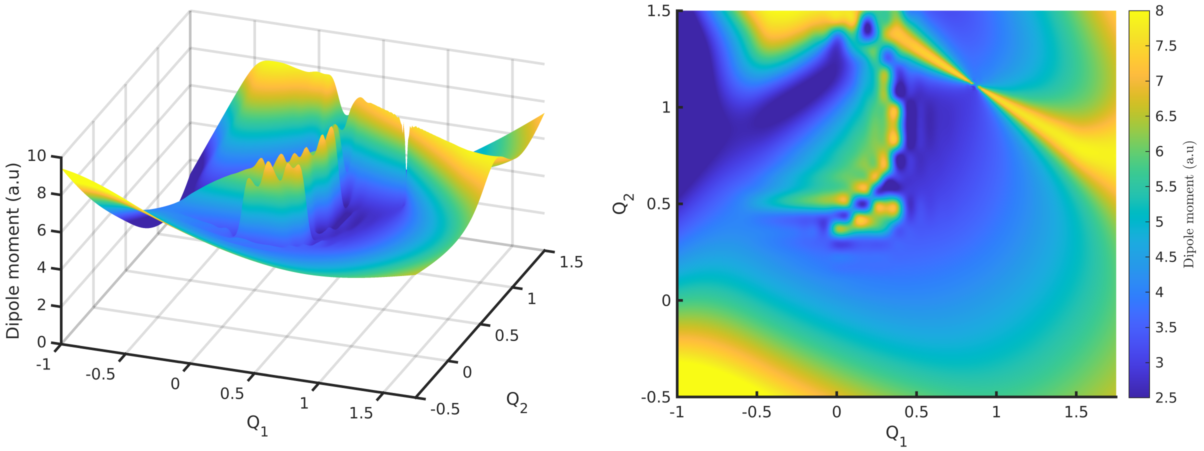

Figure S7: Permanent dipole moment of excite electronic state S_1 .

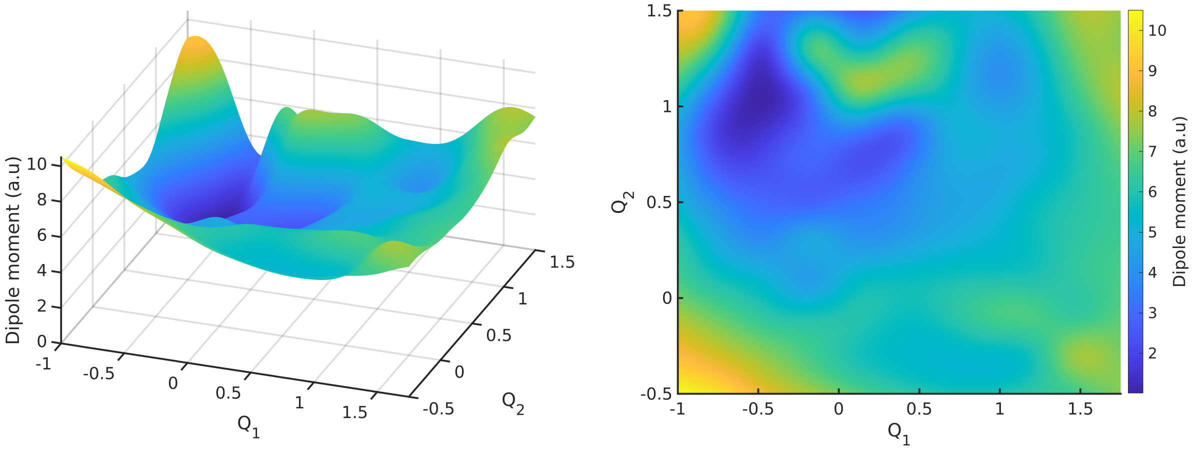

Figure S8: Permanent dipole moment of excited electronic state S_2 .

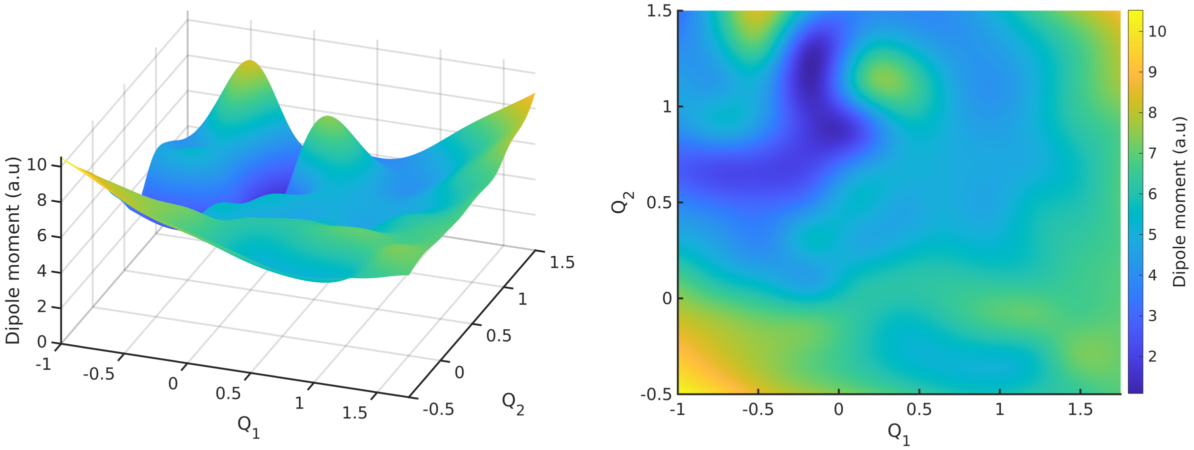

Figure S9: Permanent dipole moment of excited electronic state S_3 .

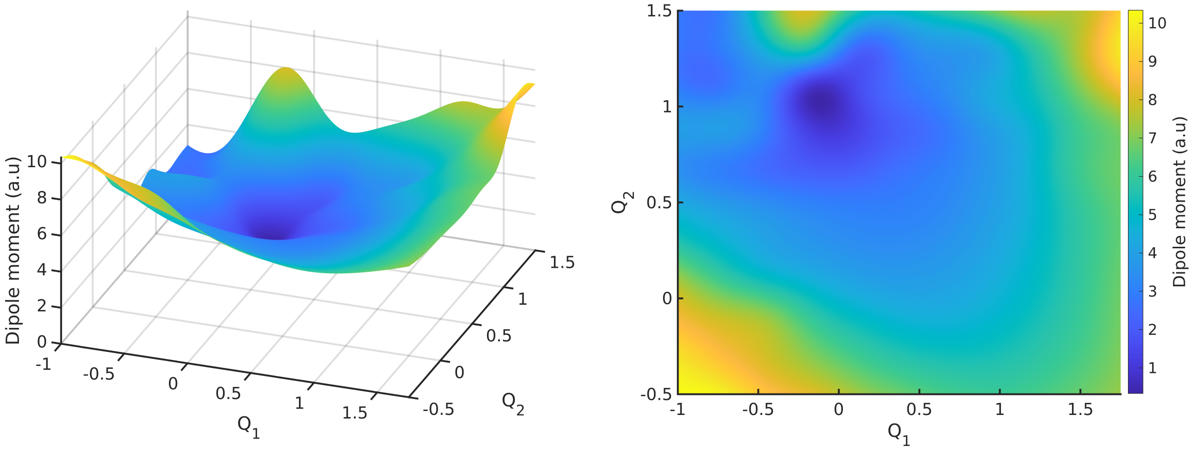

Figure S10: Permanent dipole moment of excited electronic state S_4 .

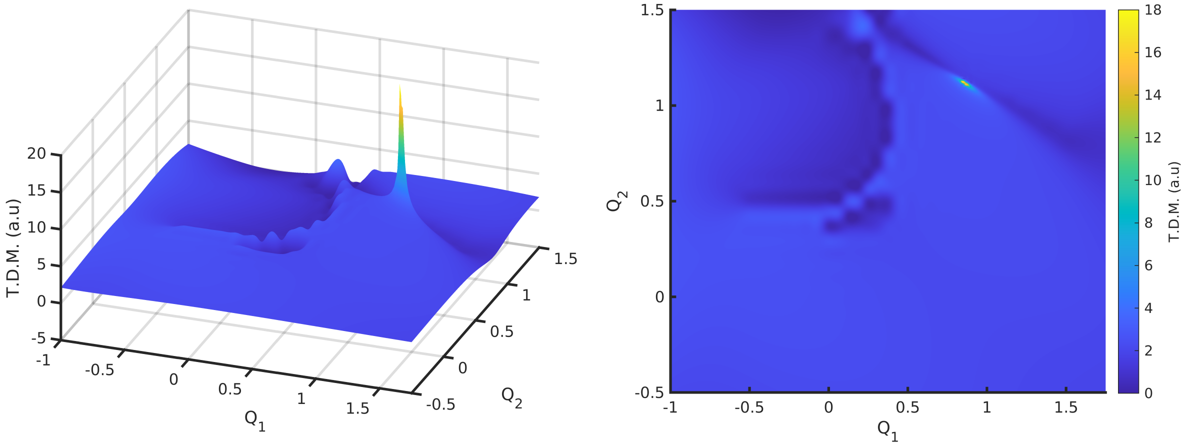

Figure S11: Transition dipole moment of the $S_0 \rightarrow S_1$.

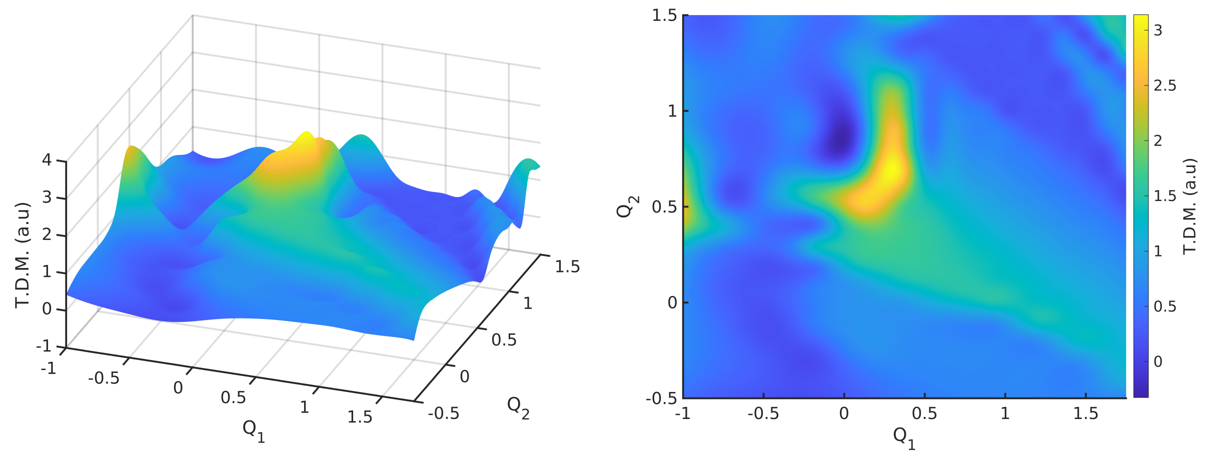

Figure S12: Transition dipole moment of the $S_0 \rightarrow S_2$.

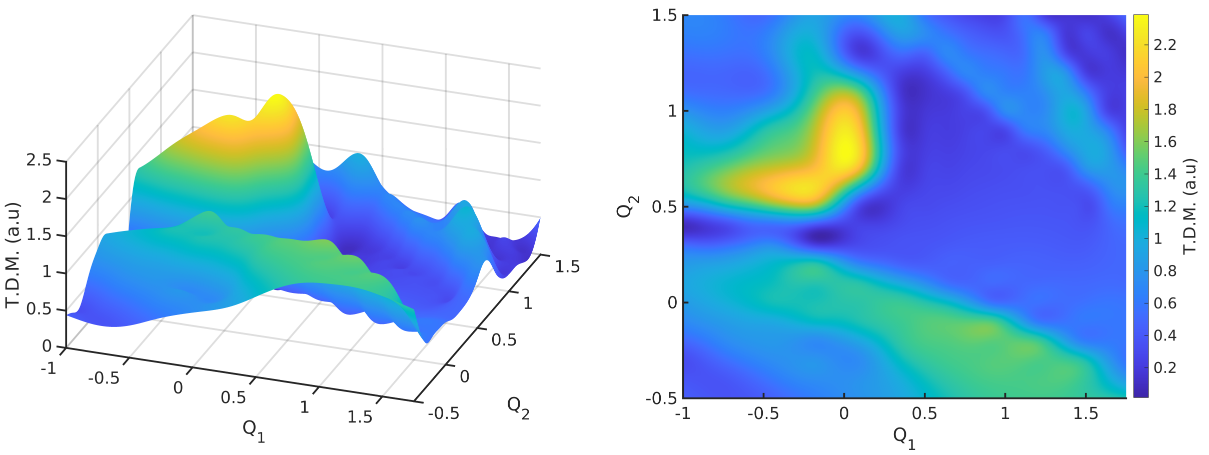

Figure S13: Transition dipole moment of the $S_0 \rightarrow S_3$.

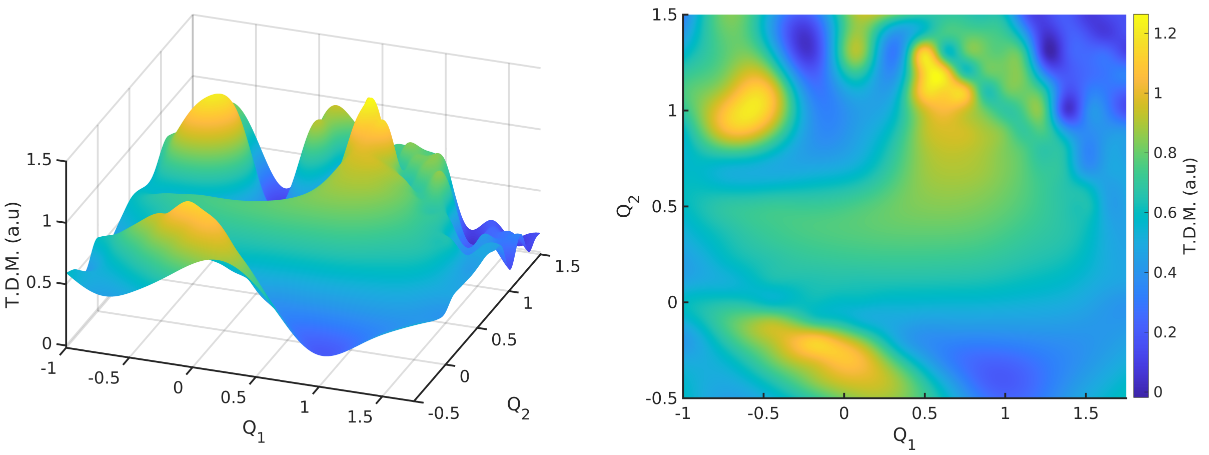

Figure S14: Transition dipole moment of the $S_0 \rightarrow S_4$.

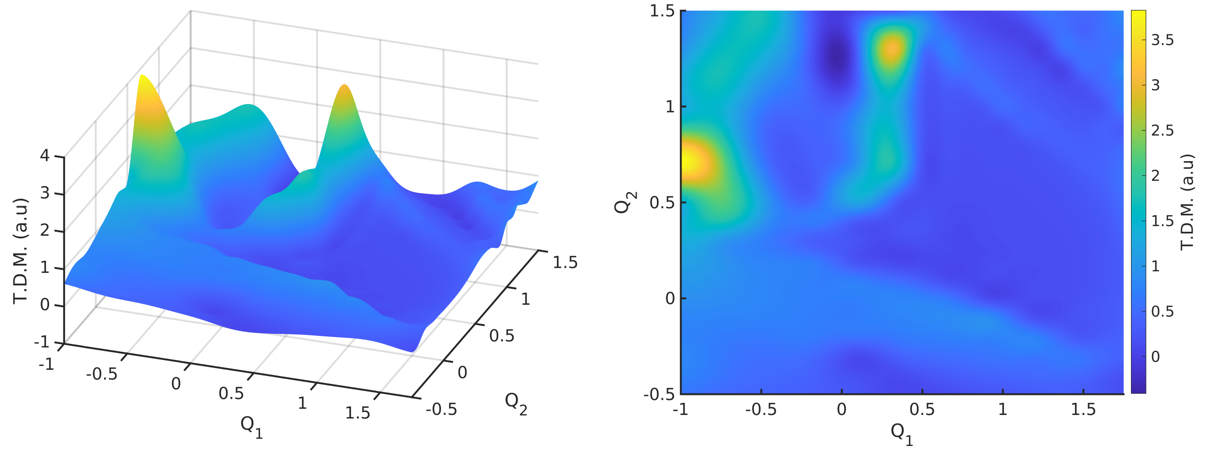

Figure S15: Transition dipole moment of the $S_1 \rightarrow S_2$.

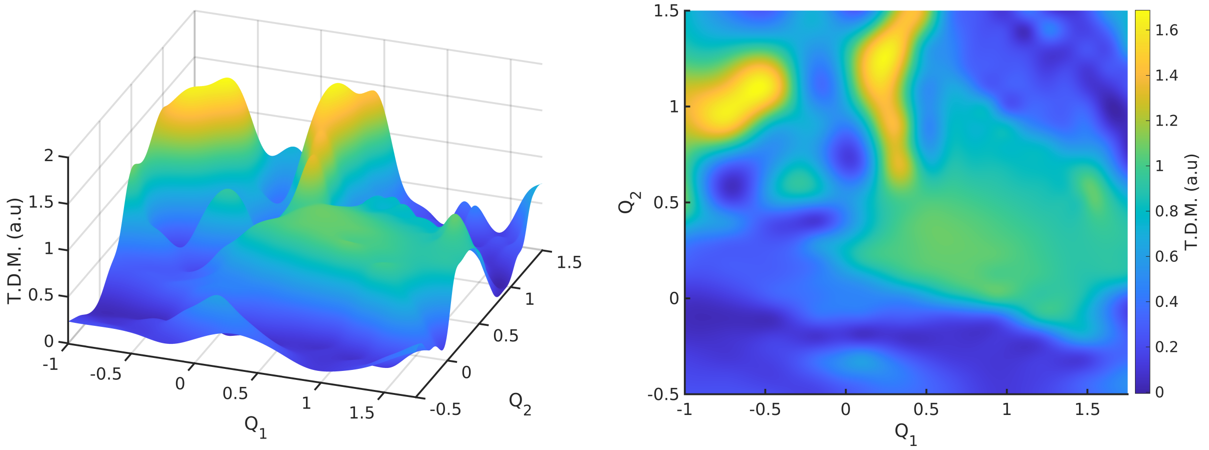

Figure S16: Transition dipole moment of the $S_1 \rightarrow S_3$.

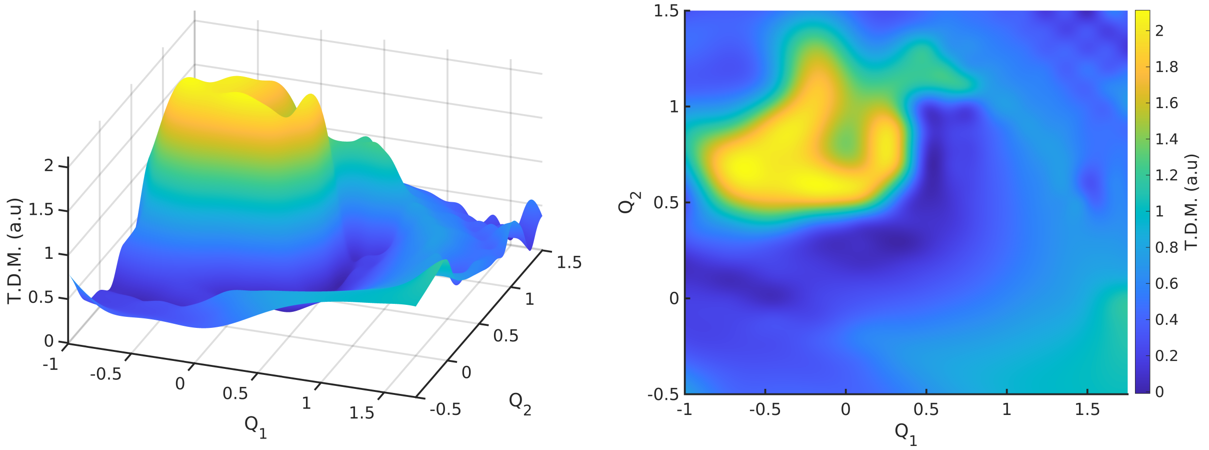

Figure S17: Transition dipole moment of the $S_1 \rightarrow S_4$.

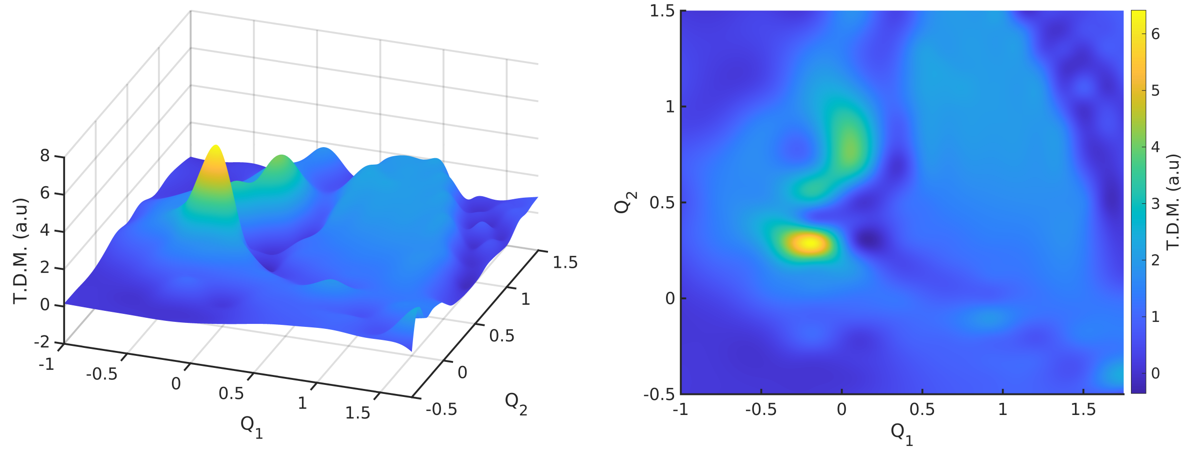

Figure S18: Transition dipole moment of the $S_2 \rightarrow S_3$.

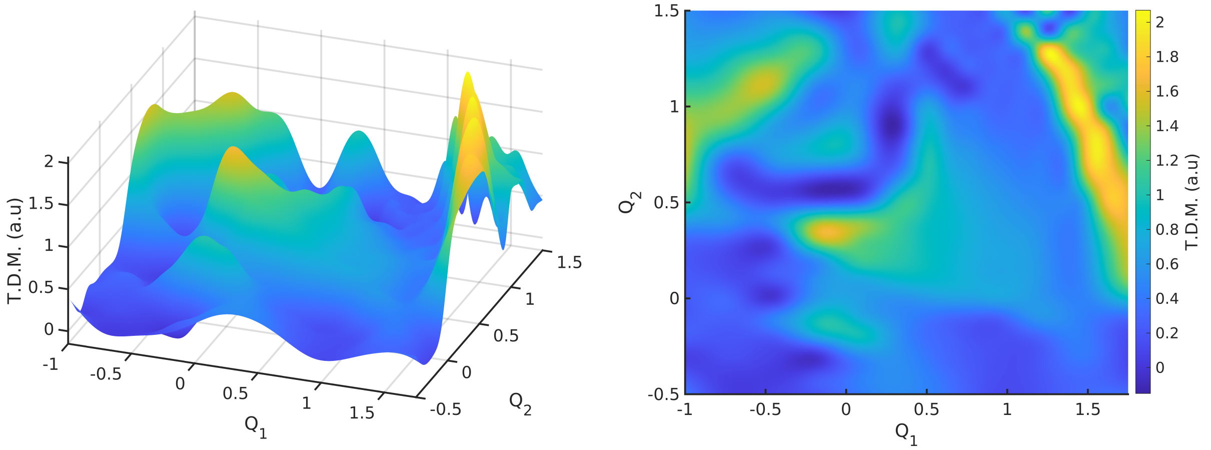

Figure S19: Transition dipole moment of the $S_2 \rightarrow S_4$.

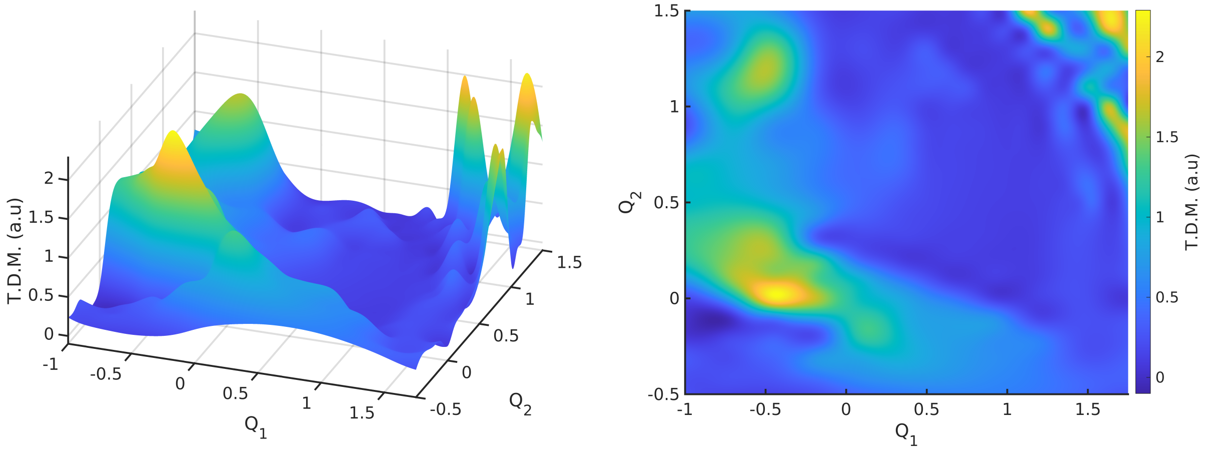

Figure S20: Transition dipole moment of the $S_3 \rightarrow S_4$.

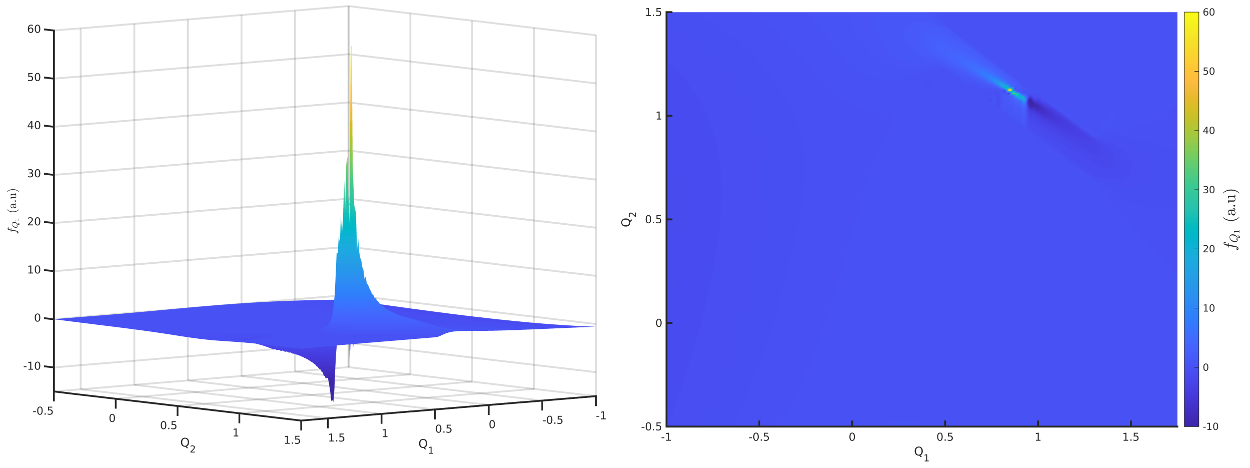

Figure S21: Non-adiabatic coupling of the Q_1 reaction coordinate.

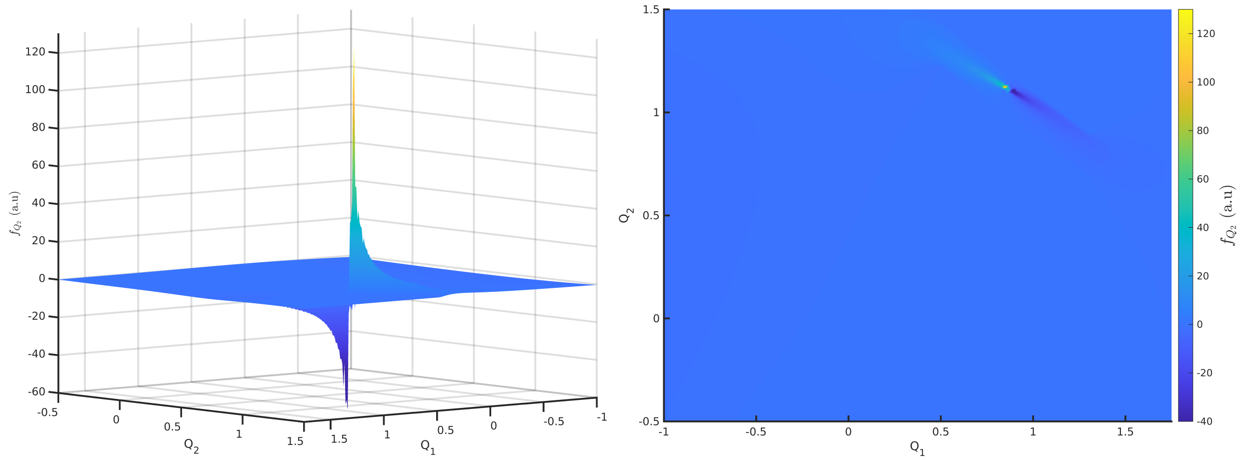

Figure S22: Non-adiabatic coupling of the Q_2 reaction coordinate.

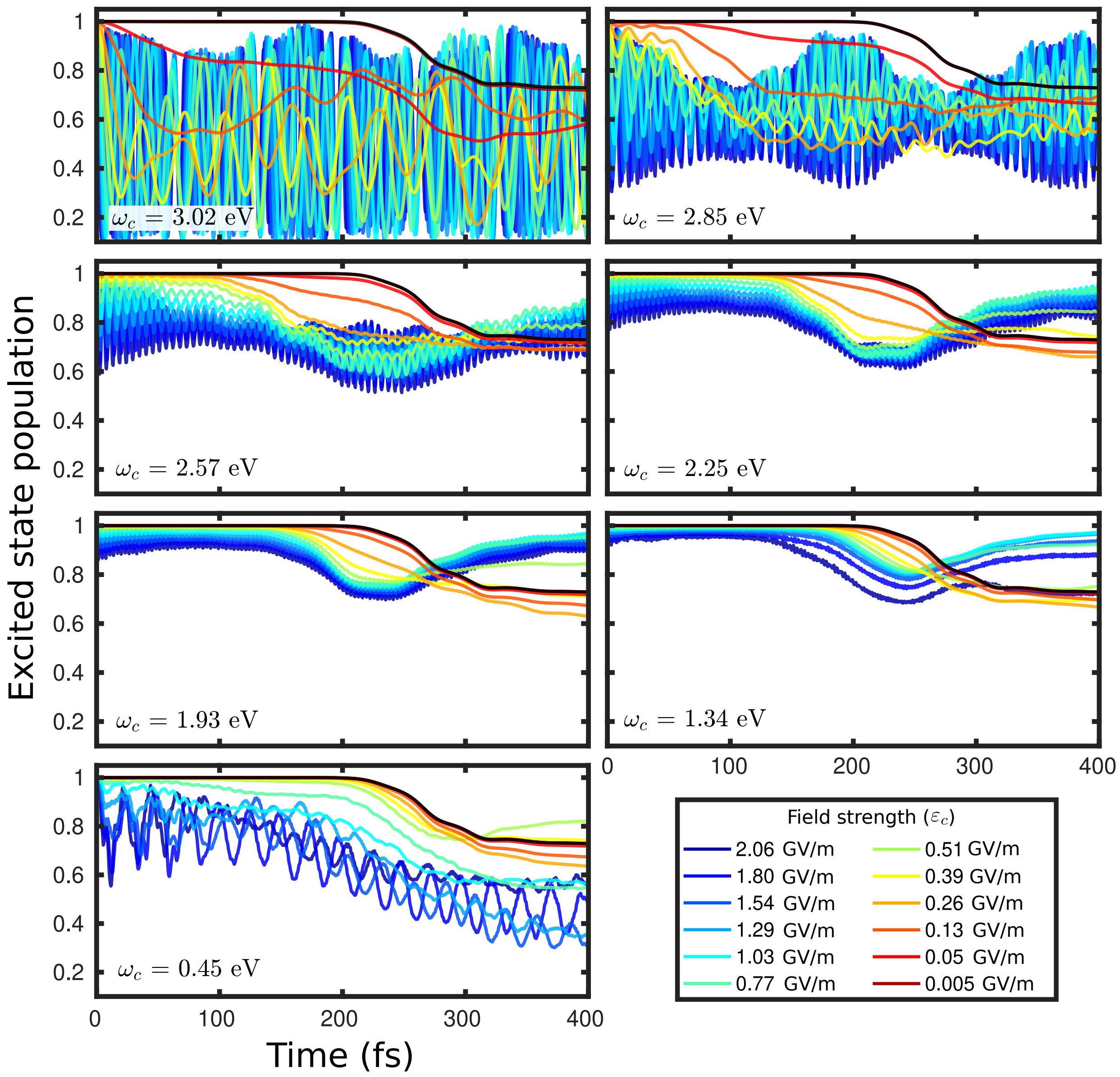

Figure S23: Time-dependent S_1 population for all values of resonance frequency ω_c and field strength ε_c discussed in the main article.

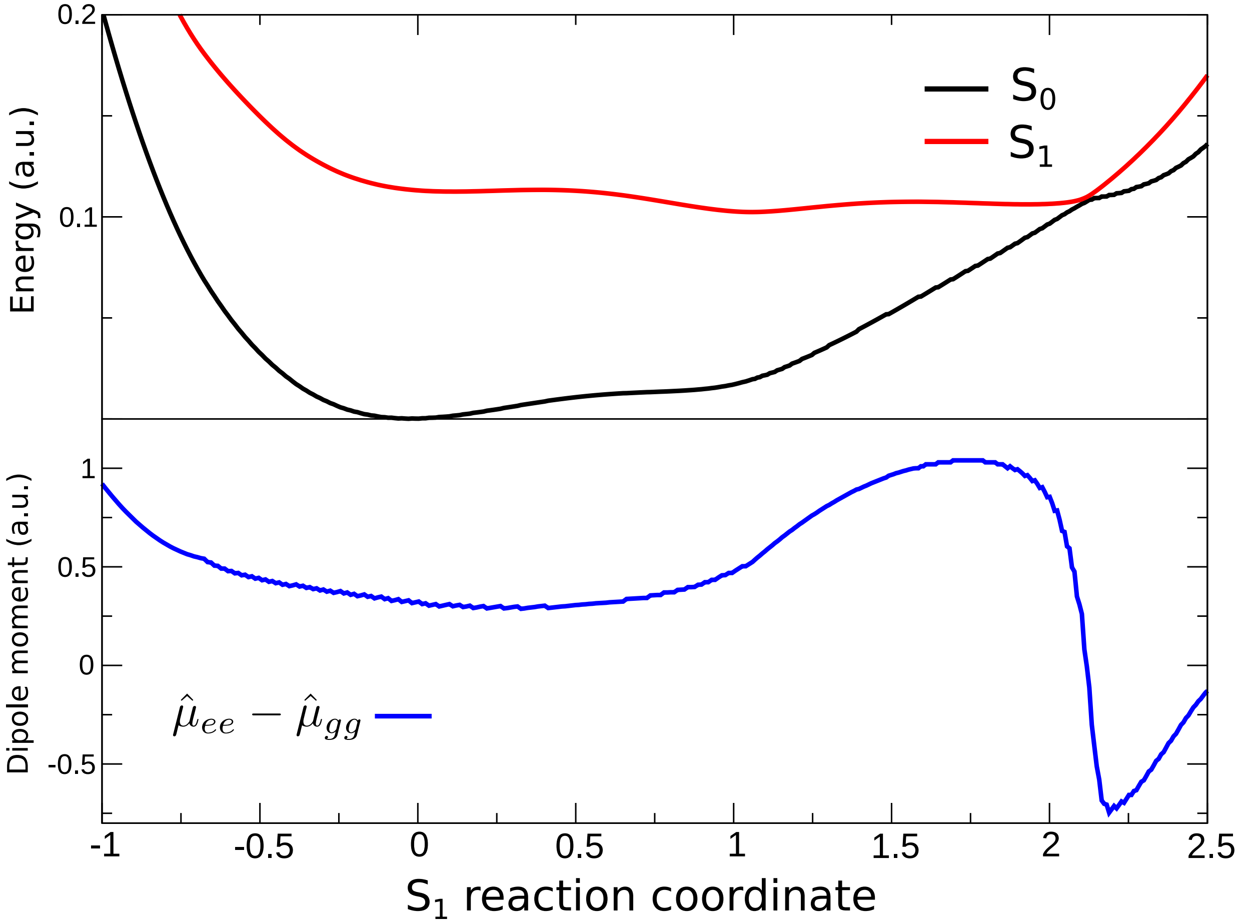

Figure S24: Potential energy curves cuts along the S_1 reaction coordinate (top panel) and difference between S_0 and S_1 dipole moments.

Photon number

Another aspect of the cavity coupled dynamics that can be analyzed is the cavity's photon number. The expectation value of the photon number operator can be written as[1]

$$\langle n \rangle = \frac{\langle H_c \rangle}{\hbar\omega_c} - \frac{1}{2}, \tag{1}$$

where H_c is the cavity Hamiltonian given by

$$\hat{H}_C = -\frac{\hbar^2}{2}\frac{\partial^2}{\partial x^2} + \frac{1}{2}\omega_c^2\hat{x}^2, \tag{2}$$

In Fig. S25 is presented the time-dependent photon number $\langle n \rangle$ from the dynamics presented in Fig. 3 of main article.

References

(1) Csehi, A.; Vibók, A.; Halász, G. J.; Kowalewski, M. Quantum control with quantum light of molecular nonadiabaticity. *Phys. Rev. A* **2019**, *100*, 053421.

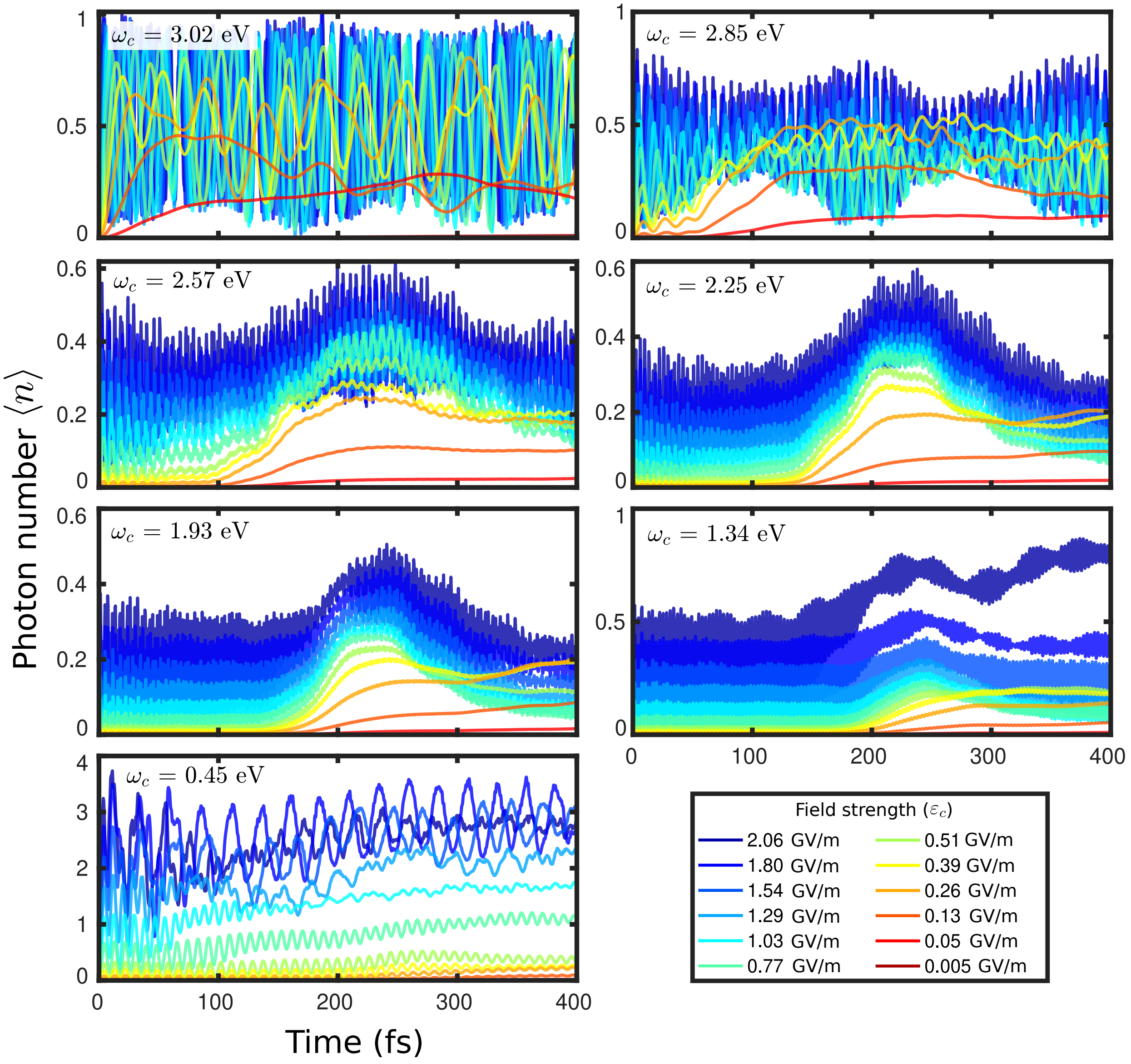

Figure S25: Time-dependent photon number from the dynamics presented in Fig. 3 of main article.

Cartesian coordinates

Ground state equilibrium (S_0^{eq}) geometry

B	2.1313376087	-0.1031074954	0.2138136088
N	1.2846108152	1.1712500545	-0.0749623626
N	1.1589593957	-1.2620914050	-0.0890476585
F	2.5072731480	-0.1184822249	1.5307275179
F	3.2151633051	-0.1435189150	-0.6197348242
C	-0.2184774483	-1.1757512400	-0.0779828090
C	-0.6984105575	-2.5123501227	-0.2267913131
C	-0.8823645329	0.0800463050	-0.0170271329
C	-2.4345525418	0.0835798549	0.0897817346
C	-0.1014860252	1.2532197643	-0.0680323724
C	-0.4054834716	2.6488171549	-0.2310299388
C	0.7821922416	3.3365599828	-0.3337585482
C	0.3958914883	-3.3513423175	-0.3276821126
C	1.8086347748	2.3752997784	-0.2229322592
C	1.5303035255	-2.5321279299	-0.2290232077
C	-3.0553538718	-0.4785947130	-1.2080778330
C	-3.0840269785	1.4650212005	0.2941826629
C	-2.8588345486	-0.7367050557	1.3313095677
H	0.9137039808	4.3888278409	-0.4771129814
H	0.3904995133	-4.4131222327	-0.4616416882
H	-2.5167428879	-1.7580353521	1.3220350710
H	-3.9416652524	-0.7487451185	1.4011548861
H	-2.4734927993	-0.2729491623	2.2338824206
H	-1.3670608775	3.0958281332	-0.2918848919

H	2.8699781764	2.5082487801	-0.2385952513
H	2.5666501019	-2.7964046895	-0.2455231318
H	-1.7155713979	-2.8269392027	-0.2778808878
H	-4.1372350954	1.3113730491	0.5001420001
H	-3.0288597583	2.0827468129	-0.5917927395
H	-2.6639666712	2.0021441899	1.1352717171
H	-4.1379333196	-0.4528960111	-1.1317163187
H	-2.7632412290	-1.4933780521	-1.4268086469
H	-2.7682918106	0.1341193385	-2.0567062769

S_1 minimum (S_1^{eq}) geometry

B	1.91938997	0.02795340	0.80552538
N	1.24230293	1.16136231	-0.00067010
N	1.17276718	-1.19992496	0.23288621
F	1.67783054	0.16768274	2.14350078
F	3.25214786	-0.04081877	0.51054943
C	-0.21920540	-1.20073831	-0.01210402
C	-0.55188553	-2.47876866	-0.49253960
C	-0.95932370	0.04003788	0.07745482
C	-2.48284535	0.10149143	0.24723232
C	-0.14844840	1.19375377	-0.25121501
C	-0.40914092	2.37039474	-0.97370798
C	0.81761149	3.03090049	-1.15730689
C	0.63421055	-3.23132866	-0.53981110
C	1.80828617	2.23451272	-0.53764595
C	1.67306289	-2.38750733	-0.08341194
C	-3.19986279	-0.02117714	-1.11149048
C	-2.89647148	1.42307491	0.92546591

C	-2.96427848	-1.02437143	1.18437135
H	0.98334465	3.96395695	-1.65472192
H	0.74289299	-4.25130634	-0.84533792
H	-2.80479883	-2.01293750	0.77837911
H	-4.02928372	-0.91530242	1.36260683
H	-2.45746694	-0.97328688	2.14235040
H	-1.35624163	2.68232019	-1.35581039
H	2.85979658	2.40425674	-0.44923553
H	2.71388548	-2.59631635	0.04055989
H	-1.51831905	-2.80475801	-0.80904288
H	-3.96075123	1.40252546	1.13684473
H	-2.70698100	2.29250567	0.31249082
H	-2.37108632	1.55773817	1.86507238
H	-4.27648619	0.03109145	-0.97731982
H	-2.97182977	-0.96266992	-1.59957813
H	-2.91067555	0.77619466	-1.78778471

Conical intersection (CoIn) geometry

N	0.90531703	1.22795232	0.15951324
B	1.25160668	0.10497800	1.20267075
N	0.86049907	-1.18297309	0.39305641
C	-0.25204115	-1.18638699	-0.43994438
C	-0.19505410	-2.34359460	-1.23756857
C	-1.13941164	-0.00642345	-0.40715605
C	-2.49320738	0.08873545	0.29568048
C	-0.20802339	1.11063225	-0.66396408
C	-0.10892235	2.08715927	-1.67191097
C	1.04796520	2.81607932	-1.41889322

C	0.93545282	-3.05233365	-0.84502092
C	1.66053007	2.22621780	-0.28090267
C	1.57537880	-2.27601747	0.15741925
F	0.46714809	0.22736534	2.31349422
F	2.58573703	0.10686355	1.47431706
H	1.42224929	3.66047634	-1.95988101
H	1.27509598	-3.99931491	-1.21059582
C	-3.60441452	0.01219471	-0.76877476
C	-2.61165528	1.41927913	1.05495402
C	-2.66457685	-1.07035087	1.28956690
H	-2.61453711	-2.03383857	0.79243240
H	-3.63235428	-1.00143681	1.77713178
H	-1.89826919	-1.04413061	2.05557008
H	-0.82137585	2.24510569	-2.45515598
H	2.58892187	2.46824781	0.19113162
H	2.49595205	-2.45685697	0.67040534
H	-0.91687789	-2.62410888	-1.97670110
H	-3.58020224	1.48415642	1.54154578
H	-2.52160876	2.26999011	0.38698660
H	-1.84512302	1.50373344	1.81644793
H	-4.58507888	0.07642872	-0.30557783
H	-3.55691290	-0.92268788	-1.31977522
H	-3.52006022	0.82540008	-1.48394527
